# Enhancing Patient-Physician Communication: Simulating African American Vernacular English in Medical Diagnostics with Large Language Models

**DOI:** 10.1007/s41666-025-00194-9

**Published:** 2025-03-11

**Authors:** Yeawon Lee, Chia-Hsuan Chang, Christopher C. Yang

**Affiliations:** 1 Drexel University, Philadelphia, PA 19104 USA; 2 Yale University, New Haven, CT 06510 USA

**Keywords:** Large language model, Health disparities, Patient simulation, Patient-physician communication gap, Communication training

## Abstract

Effective communication is crucial in reducing health disparities. However, linguistic differences, such as African American Vernacular English (AAVE), can lead to communication gaps between patients and physicians, negatively affecting care and outcomes. This study examines whether large language models (LLMs), specifically GPT-4 and Llama 3.3, can replicate AAVE in simulated clinical dialogues to improve cultural sensitivity. We tested four prompt types—BaseP, DemoP, LingP, and CompP—using United States Medical Licensing Examination (USMLE) case simulations. Statistical analyses on the models’ outputs showed a significant difference among prompt types for both GPT-4 (*F*(2,70) = 6.218, *p* = 0.003) and Llama 3.3 (*F*(2,70) = 12.124, *p* < 0.001), indicating that including demographic information and/or explicit AAVE cues influences each model’s output. Combining demographic and linguistic cues (CompP) yielded the highest mean AAVE feature counts (e.g., 9.83 for GPT-4 vs. 16.06 for Llama 3.3), although neither model fully captured the diversity of AAVE. Moreover, simply mentioning African American demographics triggers extra informal forms, suggesting built-in stereotypes or biases in both models. Overall, these findings highlight the promise of LLMs for culturally sensitive healthcare communication, while underscoring the need for continued refinement to address stereotypes and more accurately represent diverse linguistic styles.

**Supplementary Information:**

The online version contains supplementary material available at 10.1007/s41666-025-00194-9.

## Introduction

### Background

In the United States, health disparities disproportionately affect people of color and minority races, as evidenced by higher mortality rates, earlier onset of diseases, and more severe symptoms compared to the White population [1]. African Americans, for instance, face a 30% higher risk of death compared to Whites, a disparity that persists even after adjusting for age factors [2]. Conditions like hypertension, HIV, heart disease, stroke, and cancer are notably more prevalent in these minority groups. Asthma, the most common chronic childhood disease in the U.S., is 1.6 times more prevalent in Black children and 2.4 times more in Puerto Rican children compared to white children [3]. Such inequalities in health are not unique to the U.S. and can be attributed to a multitude of factors, including lifestyle differences, unequal access to medical infrastructure, structural discrimination, and stress and trauma from these conditions [4]. These factors often interact, exacerbating health issues in these communities.

While many structural, social, and economic factors contribute to these inequities, good communication between patients and providers remains a crucial yet frequently overlooked aspect. Patients who feel heard and respected are more likely to share their concerns clearly, leading to more accurate diagnoses and better treatment adherence. However, in many settings, especially among culturally and ethnically diverse groups, communication is often limited by language barriers, sociocultural differences, and deep-rooted stereotypes. These obstacles can lead to less effective healthcare interventions and poorer patient outcomes [1].

The Institute of Medicine [5] reported that disparities in treatment exist across various minority groups. For instance, Mexican Americans with myocardial infarction are 40% less likely to receive thrombolytic therapy than Whites. African Americans and Latino Americans are less likely to receive appropriate emergency care and pain management, respectively. Ortega et al. [6] found that Latino and African American children with asthma receive fewer standard management medications compared to White children.

In an increasingly global healthcare landscape, the diverse mix of cultural and linguistic backgrounds among patients creates a significant communication challenge that can widen health disparities. Effective communication in healthcare goes beyond words; it also involves understanding the different contexts in which patients experience pain and how they express it. This is where technologies like large language models (LLMs), which can understand and generate natural language, offer a novel and promising way to reduce these barriers and transform healthcare interactions and outcomes.

However, there is a caveat: if these LLMs predominantly generate responses in standard, formal English, they may not fully capture the wide range of linguistic nuances found in real-world healthcare environments. The predominance of standardized language overlooks the rich variety of linguistic styles present among patients. To begin addressing this gap, our study explores the potential of LLMs (particularly GPT-4 and Llama 3.3) to replicate diverse linguistic styles. Specifically, we investigate whether these models can emulate the linguistic characteristics of African American Vernacular English (AAVE). By evaluating their effectiveness in simulating patients with distinct linguistic traits, this research aims to bridge linguistic gaps in healthcare communication. We aspire to contribute to a more inclusive and culturally sensitive healthcare environment. This work marks the beginning of a necessary journey in healthcare technology, one with the potential to improve communication and patient outcomes.

### Prior Work

Communication barriers in healthcare are not only prevalent when providers and patients speak different primary languages [7, 8]; they also arise within the same language. Even among speakers who are native to the same language, subtle variations in intonation, speech patterns, and interaction styles [9] can lead to significant misinterpretations.

For instance, Park’s ethnographic study [10], conducted in the context of free clinics serving immigrant workers, highlights communication challenges between Korean Chinese (“Chaoxianzu” in Chinese, “Joseonjok” in Korean) migrant workers and native Korean medical staff in South Korea, despite both groups speaking Korean. Native Korean doctors often struggle to understand Korean Chinese patients. The Korean Chinese language, based on North Korea’s Munhwaŏ standard, differs significantly from South Korean in vocabulary, phonetics, phonology, stress, and intonation. Additionally, cultural differences manifest in language, particularly in how symptoms are expressed. Korean Chinese migrants often use vocabulary unrecognized by native Korean medical professionals and tend to describe symptoms in relation to specific organs. This unconventional expression can lead to unnecessary tests and confusing diagnostic processes for medical staff, while Korean Chinese patients may feel their concerns are misunderstood.

The risk of misinterpretation may be even more pronounced in the U.S., where a multitude of immigrant groups and diverse demographics coexist. Racial dynamics can further complicate communication in healthcare settings [11, 12]. Ray [13] highlights the significance of understanding historical contexts in interracial communication. In the U.S., racial minorities often aim to project competence and earn respect, while White individuals may prioritize being liked and seen as moral [14]. These differing conversational approaches can negatively impact interactions, leading to misunderstandings and adverse perceptions [15].

Linguistic variations specific to racial, demographic, or ethnic communities add another layer of complexity to communication in healthcare settings. African American Vernacular English (AAVE) is a prime example of these linguistic variations. Characterized by unique phonological, grammatical, and stylistic features, AAVE is not only a means of communication but also a powerful symbol of cultural identity for many African Americans. However, this diversity poses challenges in medical and technological contexts, as illustrated by a study [16] examining older Black adults’ interactions with voice assistants like Google Home for health information–seeking purposes. Participants, aged 50–89 from lower-income neighborhoods in Chicago and Detroit, struggled to communicate effectively with these devices. Programmed primarily in Standard English, the voice assistants failed to grasp the nuances of their dialect and accent. This language barrier necessitated a cumbersome process of cultural code-switching, rendering the interactions with the technology both cognitively demanding and time-consuming.

Such scenarios underscore a critical oversight in technology design—the failure to accommodate the rich linguistic diversity within communities. As a result, such technologies risk alienating historically marginalized groups who speak dialects like AAVE, leading to reduced long-term engagement and perpetuating digital divides in accessing health information and technology.

This issue aligns with the broader call for culturally competent healthcare, as defined by Meldrum [17]. This approach emphasizes tailoring care to each patient’s unique background, including their social, cultural, and linguistic needs. However, practical challenges in implementing such competency persist, including staff shortages and bureaucratic constraints that can impede clinicians’ ability to fully engage with patients’ linguistic and cultural diversity [4, 18, 19].

In this context, the emerging domain of Artificial Intelligence (AI), especially LLMs, offers a promising way to strengthen healthcare communication. With advanced abilities to understand and produce human-like language, these models have the potential to transform patient interactions. By capturing the subtleties of different languages and dialects, they can support more culturally responsive and language-sensitive healthcare, which can improve diagnostic accuracy and facilitate a deeper exchange of health-related information [19]. This technological progress aligns well with culturally competent healthcare goals, providing a valuable bridge over existing gaps in clinician-patient interactions.

### Goal of This Study

The primary aim of this study is to evaluate the capability of LLMs, specifically GPT-4 and Llama 3.3, to accurately simulate patients who communicate using African American Vernacular English (AAVE). AAVE, a significant variant of American English that diverges from Standard English, offers an interesting linguistic case study. Given its distinct features and status as a well-researched dialect, AAVE provides an ideal benchmark for assessing the inclusivity and accuracy of generative models’ language abilities.

Our research question is: Can LLMs, particularly GPT-4 and Llama 3.3, effectively simulate the speech of patients using AAVE? To explore this, we designed experiments in which both models took on the role of AAVE-speaking patients, answering typical diagnostic questions comparable to those asked by healthcare professionals. These patient-focused responses were generated based on the medical cases embedded in our prompts.

To identify effective prompts for simulating patients who speak AAVE, our research tests various prompt structures, from simple medical cases alone to scenarios that include additional demographic and linguistic factors. The medical cases in our prompts come from the United States Medical Licensing Examination (USMLE) Computer-Based Case Simulations (CCS), providing a diverse and realistic range of patient situations designed specifically to evaluate clinical application skills.

The feasibility of using GPT-4 and Llama 3.3 to simulate AAVE-speaking patients carries significant implications for healthcare communication. For instance, exposing clinicians to AI-generated examples of linguistic diversity, as examined in our study, can deepen their empathy and understanding of different cultural contexts. In the latter part of this paper, we explore broader applications and the potential directions for future research in more detail. Thus, the current work represents an initial step towards bridging linguistic gaps and addressing communication challenges in healthcare.

## Methods

**Fig. 1:**
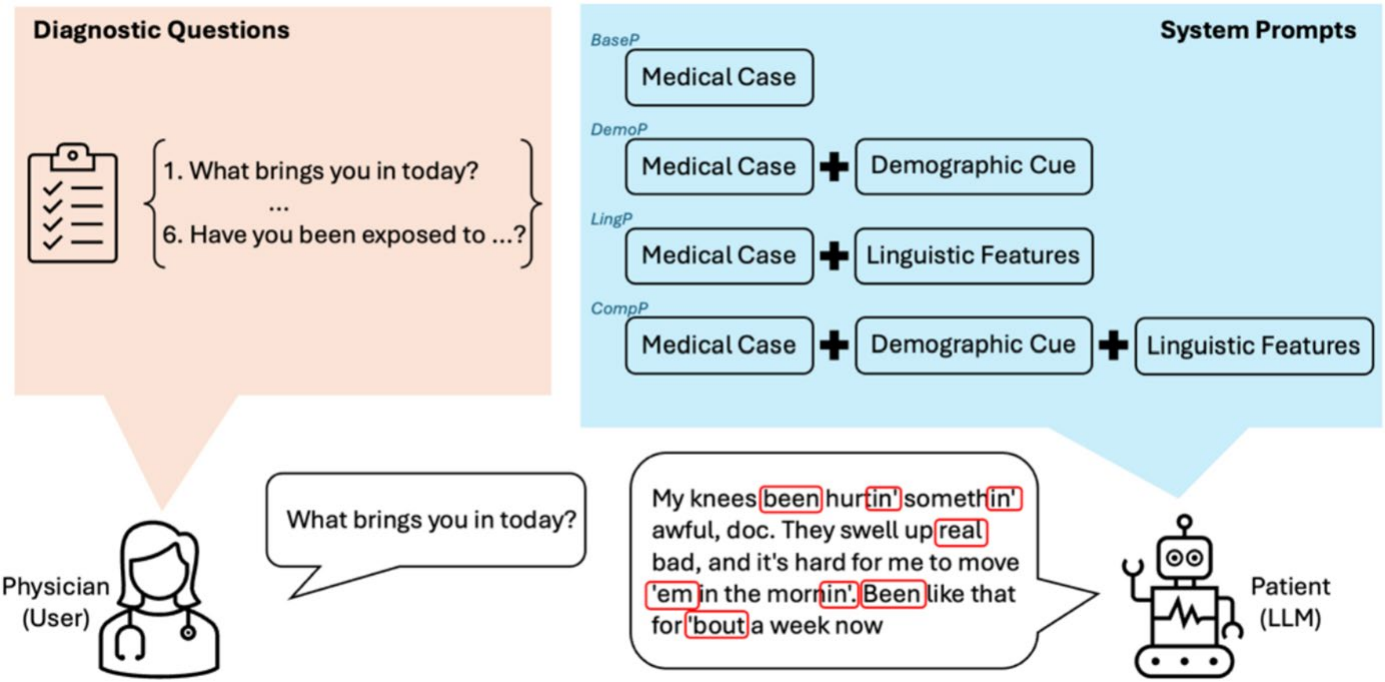
Framework for simulating patient-physician communication using LLMs

In this study, we propose a framework for simulating patient-physician communication. As shown in Fig. 1, the framework uses LLMs (GPT-4, Llama 3.3) to simulate a patient through a carefully crafted prompt. This prompt instructs the model to act as a patient within a specified medical case, incorporating a demographic cue and linguistic features. We incrementally increase the complexity by integrating these elements into each prompt. The simulated patient then engages with a set of predefined diagnostic questions prepared by ChatGPT. Lastly, we evaluate the effectiveness of the patient-physician interaction by conducting statistical analyses on the simulated patient’s responses. The following subsections detail each component of this process, covering the backbone language models, output annotation, statistical tests, and prompt elements (medical case, demographic cues, linguistic features, and questions).

### Model Details and Experimental Setup

To simulate patient-physician communication, we selected two exemplary language models: OpenAI’s GPT-4 and Meta’s Llama 3.3-70B-Instruct. These models represent advanced proprietary and open-source solutions, respectively. GPT-4, developed by OpenAI, is renowned for its advanced language understanding and generation capabilities. Conversely, Llama 3.3-70B-Instruct, an open-source model from Meta, offers solid performance with the flexibility and transparency inherent to open-source projects.

We accessed GPT-4 through OpenAI’s API and deployed Llama 3.3-70B-Instruct on four NVIDIA A40 GPUs using the vLLM [20] package to ensure efficient processing and response times. To encourage a balance between coherence and creativity in the generated dialogues, we set the temperature parameter to 0.7 for both models.

### Response Analysis

To assess the effectiveness of LLMs in simulating AAVE-speaking patients, we conducted a quantitative analysis of the linguistic features present in patient-like responses. This process involved annotating each AAVE feature and then applying statistical tests.

#### Statistical Analysis Overview

We employed ANOVA and *t*-tests on the count of annotations of AAVE features to compare different prompt types (DemoP, LingP, and CompP) and to compare GPT-4 with Llama 3.3. The goal of these analyses was to determine whether the number of identified linguistic features differed significantly across prompt types. We began with an ANOVA at *α* = 0.05 to detect any overall differences among the three prompts. Where significant effects emerged, we performed post hoc *t*-tests for pairwise comparisons (DemoP vs. LingP, DemoP vs. CompP, and LingP vs. CompP).

Because the same set of 36 responses (i.e., 36 subjects, each from the same medical case and question) was generated under every prompt condition, the design required repeated measures ANOVA and paired-samples *t*-tests. We initially examined all AAVE features as a single group, then performed the same statistical tests for each feature type individually to obtain a more detailed understanding of any observed differences.

#### Annotation Process

The annotation process was guided by an expert in AAVE, an African American healthcare professional with over 10 years of nursing experience in the U.S. She worked closely with the authors to compile a comprehensive set of AAVE linguistic features by reviewing seminal works on AAVE research and confirming these features for accuracy and cultural authenticity. Based on this review, the authors developed detailed annotation guidelines (see Appendix 1: [Annotation Guidelines]), which also include actual annotation examples illustrating how these features appear in practice. These guidelines were then used by each annotator to carry out the annotations independently.

The final label set for annotation includes the linguistic categories with bolded titles in Table 1, as well as an “out-of-list” category. The “out-of-list” category accounts for any deviations from “standard English” not covered by the specified AAVE features, regardless of whether they are unique to AAVE.

In defining the boundaries to annotate, the primary rule was to tag each word or phrase exhibiting the linguistic features outlined in Appendix 1: [Annotation Guidelines] and Appendix 2: [Linguistic features of African American Vernacular English]. Since AAVE features in the responses often overlap, the guidelines included specific rules for resolving ambiguous cases. The annotation team consisted of two experienced NLP researchers with extensive backgrounds in healthcare and biomedical domains who have worked on multiple projects involving complex annotation tasks. Label Studio [21], an open-source data-labeling tool, was used in Named Entity Recognition (NER) mode to mark relevant text spans and categorize them into predefined labels.

To examine the reliability of our annotations, we calculated the Inter-Annotator Agreement (IAA) score. Since the primary goal of this annotation process is to analyze model-generated text rather than to build a gold-standard corpus, discrepancies between annotators were not resolved procedurally. Instead, we used annotations on which two annotators reached agreement for subsequent statistical analysis. Agreement was defined as cases where both annotators classified the same text spans (with a minimum overlap of 0.8) into the same AAVE feature type.

We referred to previous study [22] for the formula to compute the degree of agreement. The following formula for positive specific agreement ($P$) is to measure the agreement between two annotators for text markup tasks.

$$P=\frac{2a}{2a+b+c}$$

where *a* is the number of features identified by both annotators, while *b* and *c* are the numbers of features identified by only one annotator.

### Prompt Design

As indicated in previous study [23], LLMs generate a response $Y=[{y}_{1},\dots ,{y}_{N}]$ based on the given context $X$. This context, often referred to as a prompt, is a natural language description of the task at hand. Let $M$ denote the LLM, which is conditioned on $X$ and generates every token ${y}_{t}$ in an autoregressive manner, $M({y}_{t+1}|X,{y}_{t})$.

In our simulated patient-physician communication, the prompts have four main components. The first is the medical case, which provides a detailed clinical scenario describing the patient’s condition or reason for visiting the doctor. The second is the demographic cue, indicating that the patient is African American and should speak in a way common to that community. The third is the set of explicit linguistic features, outlining the AAVE traits the model should include in the patient’s responses. Finally, the diagnostic questions are posed by the physician one at a time as user prompts, to which the model replies in the patient’s voice.

When setting up the conversation, the medical case and either the demographic cue or the linguistic features (or both) are combined to establish the model’s role as the AAVE-speaking patient or caregiver. Once this context is in place with the system prompt, each diagnostic question is introduced sequentially as the user prompt, and the model responds in an AAVE style that reflects the clinical scenario outlined.

To systematically assess the model’s ability to simulate an AAVE-speaking patient, we designed four types of system prompts, each with increasing complexity. We compare outputs across these variations to evaluate the impact of demographic and linguistic cues.

i. Baseline Prompt (BaseP): Delineates only the medical case, without additional demographic or linguistic variables, serving as our comparison benchmark.

**Table:** 

Your task is to role-play as a patient in the given medical case, which is enclosed by """ 1. Respond to questions posed by a user who is acting as a doctor 2. If the patient in the case cannot communicate, you should respond as their caregiver. This could be a family member or friend who has accompanied the patient 3. Always stick to the details provided in the case Medical case: """ {medical case} """

ii. Demographic Prompt (DemoP): Integrates a demographic variable with the medical case, directing the model to incorporate linguistic behavior common to the community into patient responses.

**Table:** 

Your task is to role-play as a patient in the given medical case, which is enclosed by """ 1. Respond to questions posed by a user who is acting as a doctor 2. If the patient in the case cannot communicate, you should respond as their caregiver. This could be a family member or friend who has accompanied the patient 3. Always stick to the details provided in the case 4. Ensure your responses incorporate the linguistic features common to the way many African Americans speak English Medical case: """ {medical case} """

iii. Linguistic Prompt (LingP): Combines the medical case with specific linguistic features of AAVE to evaluate the model’s capability to generate responses that explicitly adhere to detailed AAVE linguistic traits.

**Table:** 

Your task is to role-play as a patient in the given medical case, which is enclosed by """ 1. Respond to questions posed by a user who is acting as a doctor 2. If the patient in the case cannot communicate, you should respond as their caregiver. This could be a family member or friend who has accompanied the patient 3. Always stick to the details provided in the case 4. Ensure your responses incorporate the linguistic features outlined between *** Medical case: """ {medical case} """ Linguistic features: *** {features} ***

iv. Comprehensive Prompt (CompP): Intertwines the medical case with both the demographic variable and explicit linguistic traits, producing the most detailed and context-rich simulation.

**Table:** 

Your task is to role-play as a patient in the given medical case, which is enclosed by """ 1. Respond to questions posed by a user who is acting as a doctor 2. If the patient in the case cannot communicate, you should respond as their caregiver. This could be a family member or friend who has accompanied the patient 3. Always stick to the details provided in the case 4. Ensure your responses incorporate the linguistic features common to the way many African Americans speak English, as described between *** Medical case: """ {medical case} """ Linguistic features: *** {features} ***	

In the following subsections, we discuss each core component of our prompt design in greater depth. Section 2.3.1 describes how we selected and adapted the medical cases to ground our scenarios in realistic clinical contexts. Section 2.3.2 focuses on the African American demographic and the motivation for using AAVE, while Sect. 2.3.3 explores the specific linguistic features of AAVE chosen for our study. Finally, Sect. 2.3.4 explains the diagnostic questions that complete the physician–patient dialogue.

#### Medical Case

To incorporate realistic patient scenarios into our prompts, our research leveraged the USMLE, a three-step exam required for medical licensure in the U.S. Specifically, we sourced diverse medical cases from the CCS component of the Step 3. This final step of the USMLE evaluates a candidate’s ability to apply clinical knowledge and manage patient care in ambulatory settings. The CCS scenarios are designed to mimic real-world patient encounters, challenging candidates to demonstrate clinical proficiency and effective time management under time constraints.

The USMLE website [24] provides six CCS sample cases as practice material. We utilized all six cases due to their availability and open access. Each case depicts a patient with specific health conditions and contexts. Examples of these cases include a 65-year-old man with symptoms of acute chest pain and difficulty in breathing and a 32-year-old woman experiencing knee pain and swelling. The contexts include vital signs (e.g., pulse, blood pressure, and body mass index), reason for visit, history of present illness, past medical history, family history, and societal variables. For the purposes of our research, we excluded the patient’s ethnicity information, originally listed under “Identifies as,” from the medical cases. The complete text of the six medical cases can be found in Appendix 3: [Medical Cases].

#### Demographic Focus: African American and AAVE

Even in medical cases where patients have identical health conditions, the quality of patient-physician communication can vary significantly based on demographic factors such as age, gender, and race/ethnicity [15]. Our research specifically targets the African American demographic, aligning with our exploration of LLMs’ ability to simulate AAVE. AAVE, a distinct linguistic variety in the U.S., has its roots in the history of African American experiences, notably during the period of slavery [25]. Given its prevalent use and historical significance within the African American community, AAVE naturally becomes our focal demographic group. Furthermore, the existing racial disparities in U.S. healthcare between Black and White populations [26] underscore the relevance and urgency of this approach in advancing a more inclusive healthcare system. To maintain a clear focus, we deliberately exclude other demographic variables such as gender, age, geographical location, or socio-economic status. This strategy allows us to closely examine the LLM’s ability to produce linguistically relevant responses without the added complexity of multiple demographic dimensions. While our study strategically omits these factors, we acknowledge their significance and note that they represent promising directions for future research.

#### Linguistic Features of AAVE

AAVE is a dialect rich in history and unique characteristics, predominantly used by African Americans. While it is not monolithic, and indeed, just like any dialect, has regional differences in grammar, vocabulary, and pronunciation, our research necessitates a focus on certain consistent features. For the purposes of evaluating how effectively LLMs can incorporate distinct linguistic characteristics, it was essential to extract typical, common factors from AAVE to set up a baseline for evaluation. This approach allows us to assess the capability of LLMs to emulate these features systematically, even as we recognize the inherent variability among speakers and regions.

Its origins can be traced back to Southern American English, a connection highlighted by Labov [27]. This dialect evolved from the English variants spoken by African slaves and their descendants, predominantly located in the Southern United States. Labov’s analysis of AAVE in relation to other dialects reveals notable similarities with Southern White dialects, particularly in syntax elements like negative concord—the use of multiple negatives to express a single negation—and double modals, which involves the use of two modal verbs together.

Rickford’s [28] foundational research offers a comprehensive compilation of AAVE features, establishing it as a key reference point for subsequent studies. He detailed unique traits in AAVE’s phonology and grammar. Grammatically, AAVE is distinguished from Standard English by its treatment of verb tense, aspect, and mood, as well as its use of pronouns and negation. Notably, the omission of the copula/auxiliary “is” and “are” in present tense leads to constructions like “He tall” instead of “He is tall.” AAVE also employs the invariant “be” to indicate habitual aspects, as in “He be walkin,” and uses unstressed “been” or “bin” for what in Standard English would be “has/have been.” A stressed “bin” denotes actions or states that commenced long ago and may still continue. The use of “done” in AAVE emphasizes completed actions and can co-occur with “been,” as in “He done did it” or “They done been sitting there an hour.” In terms of nouns and pronouns, AAVE often omits the possessive -s, as in “John house,” and can use associative plurals marked by “and (th)em” or “nem.” Negation in AAVE, characterized by the use of “ain’t” as a general pre-verbal negator and multiple negation or negative concord, is a well-studied aspect as well.

Pullum [29] complements the understanding of AAVE by providing more nuanced rules in negative concord and copula omission. Pullum notes the repositioning of negative auxiliary verbs at sentence beginnings, especially when the subject is indefinite, as in “Ain’t nobody gonna find out.” He also enumerated contexts where copula omission does not occur, such as when the copula bears accent, is infinitival, expresses habitual aspect, is in the past tense, is first-person singular, begins a clause, or occurs in a confirmatory tag at the end of a sentence.

Building upon these foundational studies, Wolfram [30] and Thomas [31] add social, cultural, and economic dimensions to our understanding of AAVE. Thomas focuses on the unique migration history of AAVE, originating in the South and transitioning to urban centers during the Great Migration. This shift to urban life significantly influenced the dialect, leading to some dialect leveling as African Americans from various regions mixed in new urban communities. Wolfram highlights AAVE’s strong association with urban black youth culture, noting the age-graded usage of “habitual be,” predominantly found among younger speakers. This suggests a continuous evolution of AAVE within urban settings. Wolfram identifies a “supra-regional core” of AAVE, acknowledging some regional variation but emphasizing shared features across different urban areas. These key grammatical features mostly align with Rickford’s work and include copula absence, invariant BE, completive “be done,” remote “been,” and unique traits in negation and nominals. Wolfram also categorizes the features of urban AAVE into stable, intensifying, and receding traits, highlighting the dynamic nature of the dialect. This emphasizes the continuity with historical rural AAVE roots and the ongoing changes reflecting urban influences.

In this research, while recognizing the dynamic nature and diversity within AAVE, as explored by these works, we primarily draw upon Rickford’s presentation of AAVE. His works offers clarity, comprehensive coverage, and foundational status in AAVE studies. However, we have tailored his framework to align with our research objectives. For instance, we excluded many phonological aspects due to the limitations of LLMs, which produce written responses and cannot capture phonetic aspects. Additionally, to prevent the LLMs from merely replicating specific vocabulary, we have minimized the inclusion of direct vocabulary presented as lexical features in Rickford’s work. Nonetheless, we selectively incorporate certain phonological and lexical features that are central to AAVE and widely discussed in the literature. Our methodology also omits features less frequently mentioned in recent studies, focusing on the most prominent and impactful aspects of AAVE as per current academic consensus. This approach acknowledges the limitations of our study, particularly in the context of phonological representation, while striving for a comprehensive and relevant analysis of AAVE within the capabilities of current LLMs.

Ultimately, our research focuses on 37 carefully selected features. Table 1 presents the linguistic features identified for our research, with a descriptive profile for each feature on the right and their corresponding categories on the left. This categorization was important for effectively identifying and annotating AAVE features in the patients’ responses. Utilizing all 37 features as separate labels would have resulted in an impractically extensive list for annotation purposes. For a more practical approach, we decided to use only the bolded titles in Table 1 as labels, making it easier to identify AAVE features in the patients’ responses.

**Table 1:** AAVE linguistic features

Grammatical features	Pre-verbal markers	Omission of “is” and “are”	
		Invariant “be”	habitual actions
			contractions of “will/would be”
		“been”/ “bin”	unstressed “been” for present perfect
			stressed “been” for the action that happened a long time ago
		Use of “done” for a distant past tense	
		Use of “be done” for a future perfect tense	
		Use of “had” for a past tense	
		Use of double modals	
	Verbal tense-number marking	Absence of third person singular present -s, doesn’t, or has	
		Use of “is” and “was” for plural and second person subjects	
		Use of past tense for past participle	
		Use of past participle for past tense	
		Use of verb stem (root forms) for past tense	
		Reduplicated Tense Marking	
	Nouns and pronouns	Unmarked possessives	
		Unmarked plural forms	
		Regularization of irregular plural nouns	
		Use of “an ‘em,” “and ‘em,” “nem” to mark associative plurals	
		Appositive or pleonastic pronouns	
		Use of “y’all” for the 2nd person plural	
		Use of demonstrative “them”	
		Omission of relative pronoun	
	Negation	Negative concord	
		Negative inversion	
		Use of “ain’t” as a general pre-verbal negator	
		Use of “ain’t” + “but,” and “don’t” + “but” to indicate “only”	
	Questions	Formation of direct questions without inversion	
		Inversion in embedded questions	
	Existential-locative construction	Use of existential “it,” “they” (or “dey”)	
		Use of existential “they got”	
		Use of “here go” as a static locative or presentational form	
Lexical features		Use of “steady” for consistent, persistent or repeated action	
		Use of “come” to imply the speaker’s indignation	
		Use of “finna” to indicate immediate future actions	
Phonological features		Replacement of final “ing” with “in’”	
		Omission of unstressed syllables at the beginning and middle	

While we have tried to compile a thorough list of these linguistic features, the list is not exhaustive. Therefore, we introduced an “Out-of-list” label to capture instances of non-standard usage that are not explicitly included in the literature-based AAVE feature sets but remain relevant to our study. These might be AAVE features not captured by our research, non-standard forms not previously documented in AAVE studies, or simply generalized informal speech.

In Appendix 2, detailed explanations and examples are provided for all features except out-of-list features.

#### Questions for Diagnostic Interaction

The simulation of patient-physician interactions is conducted by sequentially posing a set of prepared questions to the LLM. These diagnostic questions, crafted by ChatGPT in response to the query, “common questions physicians ask patients,” are designed to maintain neutrality concerning the patient’s gender, age, and symptoms. The selection of these questions is strategically based on their prevalence and universal relevance across all six medical cases, ensuring that they are broadly applicable and fundamental to the diagnostic process. As a result, we have compiled a list of six diagnostic questions routinely used by physicians:

1. What brings you in today?
2. Have you had any procedures or major illnesses in the past 12 months?
3. Are you currently taking any medications, including over-the-counter and herbal supplements?
4. What allergies do you have?
5. Have you traveled anywhere recently?
6. Have you been exposed to anyone who's been sick recently?

Additionally, to accommodate scenarios where direct responses from patients may not be possible, such as in cases involving young children or patients in coma, we adapted the phrasing of each question, creating variations of the questions with different subjects (“you,” “he,” “she”). This allows the simulation to accommodate a wide range of clinical scenarios, promoting an uninterrupted and natural flow of conversation throughout the diagnostic interaction.

Concluding this section, the algorithm below provides a concise overview of our simulation workflow, illustrating how each medical case, prompt type (BaseP, DemoP, LingP, or CompP), and set of AAVE features are combined before diagnostic questions are fed to the model. The resulting outputs are then collected for subsequent annotation and analysis. Having detailed our methodological approach, we now turn to Sect. 3 to discuss the results that emerged from these simulations.

**Algorithm:**
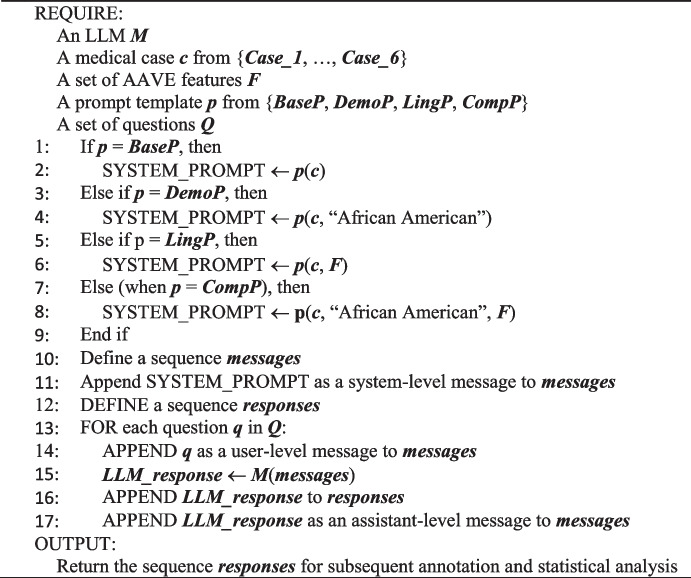
Patient-Physician Dialogue Simulation

## Results

The subsequent sections provide a quantitative analysis of the AAVE features identified by both annotators, starting with the annotation result.

### Annotation

Table 2 quantitatively demonstrates the consistency of agreement among annotators in annotating linguistic features. The inter-annotator agreement (IAA) for GPT-4’s outputs is 0.899 overall, while Llama 3.3 achieves a slightly higher 0.942, indicating substantial agreement in both cases. Most grammatical and phonological features consistently show high reliability, suggesting that both LLMs produce clearly identifiable patterns of AAVE.

**Table 2:** Inter-annotator agreement for each linguistic feature

			*a*	*b*	*c*	*P*
GPT-4	Grammatical Features	Pre-verbal markers	155	3	36	0.888
		Verbal tense-number marking	15	1	3	0.882
		Nouns and pronouns	14	6	4	0.737
		Negation	298	14	3	0.972
		Questions	0	0	0	-
		Existential-locative construction	0	0	0	-
	Lexical Features		1	0	1	0.667
	Phonological Features		186	1	11	0.969
	Out-of-List		234	90	31	0.795
	Total		903	115	89	0.899
			*a*	*b*	*c*	*P*
Llama 3.3	Grammatical Features	Pre-verbal markers	238	10	7	0.966
		Verbal tense-number marking	56	7	6	0.896
		Nouns and pronouns	73	5	5	0.936
		Negation	232	6	5	0.977
		Questions	0	0	0	-
		Existential-locative construction	0	0	0	-
	Lexical Features		11	0	0	1.000
	Phonological Features		694	16	14	0.979
	Out-of-List		183	90	12	0.782
	Total		1487	134	49	0.942

*a* is the number of features both annotators agreed on, and *b* and *c* are features identified by only one annotator. *P* (positive specific agreement) measures how often two annotators identify the same features. For the formula for *P*, refer to Sect. 2.2.2

However, lexical features represent a notable exception. Specifically, in the case of GPT-4, over the phrase “This just come on real sudden,” one annotator identified “come” as a lexical feature conveying annoyance or anger, while the other annotator classified it under verbal tense–number marking because it omits the third person “-s.” Because we did not conduct a formal disagreement-resolution process, these borderline examples remained unaligned, resulting in lower IAA for certain features. Overall, these findings suggest that although annotators reliably capture the core grammatical aspects of AAVE, they tend to disagree on less frequent or more context-dependent features such as specific lexical markers. All subsequent statistical analyses in this section were based on the annotations where both annotators agreed.

### Comparison of Prompt Effectiveness

We assessed the effectiveness of various prompts by counting the number of AAVE linguistic features. Because BaseP, which serves as a baseline without any demographic or linguistic information, did not yield any AAVE linguistic features, we excluded it from this analysis. Our focus was on comparing the effectiveness of DemoP, LingP, and CompP, aiming to determine which prompt most frequently elicited AAVE linguistic features in the simulated patients.

**Fig. 2:**
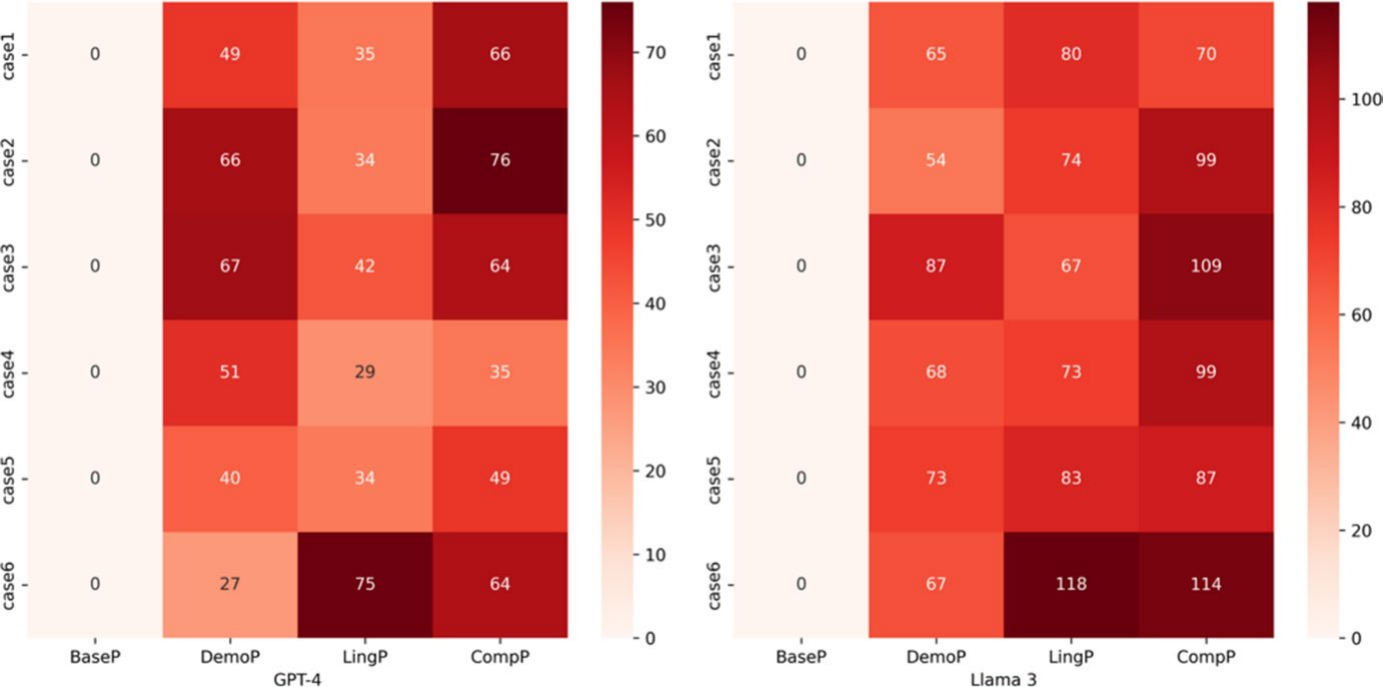
Distribution of AAVE feature annotations by diagnostic case

Figure 2 presents two heatmaps that compare GPT-4 and Llama 3.3 on six medical cases (Case1 to Case6), each of which consists of six responses respectively, arranged by prompt type (BaseP, DemoP, LingP, and CompP). The color in each cell reflects how many AAVE features appear in that case. Overall, CompP tends to produce more AAVE features than DemoP and LingP for both models. However, in GPT-4, DemoP (which includes the demographic variable) generally yields higher counts of AAVE features than LingP (which includes a list of AAVE linguistic features). By contrast, with Llama 3.3, LingP tends to produce more features than DemoP.

Table 3 shows both descriptive statistics and ANOVA results for all feature types across the three prompt conditions. Each condition contained 36 data points, which reflect six different medical cases. For each case, six “patient” responses were provided by the LLM, giving a total of 36 observations per condition.

**Table 3:** One-way repeated measures ANOVA for all types

	GPT-4			Llama 3.3		
	Count	Sum	Mean (SD)	Count	Sum	Mean (SD)
DemoP	36	300	8.333 (3.964)	36	414	11.500 (3.418)
LingP	36	249	6.917 (4.211)	36	495	13.750 (4.753)
CompP	36	354	9.833 (4.411)	36	578	16.056 (5.909)
	Sum of squares	*F*(2, 70)	*p*-value	Sum of squares	*F*(2, 70)	*p*-value
Condition	153.167	6.218	0.003	373.574	12.124	< 0.001
Error	862.167			1078.426		

GPT-4 demonstrated mean scores of 8.33 with a standard deviation of 3.96 for DemoP, 6.92 with a standard deviation of 4.21 for LingP, and 9.83 with a standard deviation of 4.41 for CompP. A one-way repeated measures ANOVA revealed a statistically significant difference among these three conditions for GPT-4, with a sum of squares of 153.167, *F*(2,70) = 6.218, and *p* = 0.003. Similarly, Llama 3.3 exhibited mean scores of 11.50 (SD = 3.42) for DemoP, 13.75 (SD = 4.75) for LingP, and 16.06 (SD = 5.91) for CompP, also with 36 observations in each condition. Its one-way repeated measures ANOVA indicated a significant effect of condition, with a sum of squares of 373.574, *F*(2,70) = 12.124, and *p* < 0.001.

These results show that both GPT-4 and Llama 3.3 respond differently across the three prompt types, indicating that changes in prompt content (demographic information, explicit AAVE cues, or both) influence the features they generate. Given the significant ANOVA findings, the next step is to examine pairwise comparisons using paired *t*-tests.

Table 4 presents the results of *t*-tests on pairs of prompts to pinpoint differences among DemoP, LingP, and CompP. Although we initially expected CompP to yield more features, since it offers the most comprehensive cues about AAVE, we had no clear prediction about how DemoP and LingP might compare. Therefore, we used two-tailed tests and differences were calculated as Group 2 minus Group 1 for each pair. Results of one-tailed tests are available in Appendix 4: [Comprehensive Statistical Results].

**Table 4:** Paired* t*-test for all types

	GPT-4			Llama 3.3		
Group1	DemoP	DemoP	LingP	DemoP	DemoP	LingP
Group2	LingP	CompP	CompP	LingP	CompP	CompP
Mean	− 1.417	1.500	2.917	2.250	4.556	2.306
SD	4.965	4.681	5.228	5.432	5.739	5.476
SE	0.827	0.780	0.871	0.905	0.957	0.913
*t*-test (df = 35)	− 1.712	1.923	3.347	2.485	4.762	2.526
*p* (2-tail)	0.096	0.063	0.002	0.018	< 0.001	0.016

Based on the two-tailed *p*-values, GPT-4 showed a statistically significant difference only for the LingP vs. CompP comparison (*p* = 0.002). In contrast, the DemoP vs. LingP (*p* = 0.096) and DemoP vs. CompP (*p* = 0.063) comparisons did not reach the 5% significance threshold. This indicates that including both demographic information and AAVE linguistic cues (CompP) yields a significantly different outcome than using only AAVE linguistic cues (LingP). Simply adding demographic information (DemoP) rather than linguistic cues (LingP) was not clearly significant, and demographic-only (DemoP) vs. combined (CompP) was borderline.

For Llama 3.3, however, all three pairwise comparisons were statistically significant, with *p*-values below 0.05. This suggests that Llama 3.3’s performance varies more noticeably among the three prompts, whereas GPT-4 shows a clear difference only between LingP and CompP. In other words, any adjustment you make, such as adding demographic cues, adding AAVE linguistic cues, or combining both, changes Llama 3.3’s behavior significantly.

We conducted fine-grained analyses for each specific linguistic feature type. Table 5 shows how nine linguistic feature types are distributed among the three prompt categories DemoP, LingP, and CompP for both GPT-4 and Llama 3.3.

**Table 5:** Distribution of linguistic feature types across prompts

	GPT-4			Llama 3.3		
	DemoP	LingP	CompP	DemoP	LingP	CompP
Pre-verbal markers	35	52	68	19	119	100
Verbal tense-number marking	7	3	5	5	28	23
Nouns and pronouns	1	3	10	17	25	31
Negation	101	84	113	64	78	90
Questions	0	0	0	0	0	0
Existential-locative construction	0	0	0	0	0	0
Lexical features	1	0	0	0	4	7
Phonological features	68	32	86	245	200	249
Out-of-list	87	75	72	64	41	78
Total	300	249	354	414	495	578

Notably, Llama 3.3 produces more features overall, as shown by the higher total counts for each prompt type (414, 495, and 578) compared to GPT-4 (300, 249, and 354). For example, Llama 3.3 generates between 200 and 249 phonological features across the three prompt types, whereas GPT-4’s counts range from 32 to 86. Llama 3.3 also produces substantially more pre-verbal markers, especially in the LingP (119) and CompP (100) conditions, while GPT-4’s output for the same feature ranges more moderately between 35 and 68. Verbal tense-number marking follows a similar trend and appears far more frequently in Llama 3.3’s responses, with counts of 28 for LingP and 23 for CompP, compared to GPT-4’s 3 and 5.

By contrast, GPT-4 makes greater use of negation, especially under DemoP and CompP (101 and 113), compared to Llama 3.3’s counts of 64 and 90. Another notable difference is GPT-4’s minimal or nonexistent usage of lexical features (1 or 0 occurrences), whereas Llama 3.3 produces a handful, particularly in the CompP condition (7).

Neither model employs existential-locative constructions or question forms in these experimental settings (both show zero occurrences), suggesting that certain AAVE markers remain underrepresented in their simulated responses. Since these two features did not appear in either model, further per-feature statistical analysis (ANOVA and post hoc *t*-tests) focused on the remaining seven features. Comprehensive statistical tables—including detailed descriptive statistics, full ANOVA and *t*-tests results for each feature type, and one-tailed *p*-values—are provided in Appendix 4: [Comprehensive Statistical Results].

Tables 6, 7, 8, 9, 10, 11, and 12 present a feature-by-feature statistical summary of GPT-4 and Llama 3.3, organized by seven feature types.

**Table 6:** Per-type analysis. Pre-verbal markers

		GPT-4			Llama 3.3		
Pre-verbal markers	ANOVA	Mean (SD)	*F* (2, 70)	*p*	Mean (SD)	*F* (2, 70)	*p*
	DemoP	0.972 (1.028)	7.334	0.001	0.528 (0.941)	22.477	< 0.001
	LingP	1.444 (1.229)			3.306 (2.638)		
	CompP	1.889 (1.753)			2.778 (2.231)		
	*t*-test	Mean (SD)	*t*-test (df = 35)	*p* (2-tail)	Mean (SD)	*t*-test (df = 35)	*p* (2-tail)
	LingP-DemoP	0.472 (1.230)	2.303	0.027	2.778 (2.819)	5.911	< 0.001
	CompP-DemoP	0.917 (1.461)	3.763	< 0.001	2.250 (2.465)	5.476	< 0.001
	CompP-LingP	0.444 (1.594)	1.673	0.103	− 0.528 (2.624)	− 1.207	0.236

**Table 7:** Per-type analysis. Verbal tense-number marking

		GPT-4			Llama 3.3		
Verbal tense-number marking	ANOVA	Mean (SD)	*F* (2, 70)	*p*	Mean (SD)	*F* (2, 70)	*p*
	DemoP	0.194 (0.401)	1.094	0.341	0.139 (0.424)	10.209	< 0.001
	LingP	0.083 (0.280)			0.778 (0.898)		
	CompP	0.139 (0.424)			0.639 (0.931)		
	*t*-test	Mean (SD)	*t*-test (df = 35)	*p* (2-tail)	Mean (SD)	*t*-test (df = 35)	*p* (2-tail)
	LingP-DemoP	− 0.111 (0.465)	− 1.435	0.160	0.639 (0.723)	5.301	< 0.001
	CompP-DemoP	− 0.056 (0.475)	− 0.702	0.487	0.500 (0.971)	3.090	0.004
	CompP-LingP	0.056 (0.410)	0.813	0.422	− 0.139 (0.961)	− 0.867	0.392

**Table 8:** Per-type analysis. Nouns and pronoun

		GPT-4			Llama 3.3		
Nouns and pronouns	ANOVA	Mean (SD)	*F* (2, 70)	*p*	Mean (SD)	*F* (2, 70)	*p*
	DemoP	0.028 (0.167)	4.958	0.010	0.472 (0.736)	2.575	0.083
	LingP	0.083 (0.368)			0.694 (0.920)		
	CompP	0.278 (0.513)			0.861 (0.990)		
	*t*-test	Mean (SD)	*t*-test (df = 35)	*p* (2-tail)	Mean (SD)	*t*-test (df = 35)	*p* (2-tail)
	LingP-DemoP	0.056 (0.410)	0.813	0.422	0.222 (1.017)	1.311	0.199
	CompP-DemoP	0.250 (0.500)	3.000	0.005	0.389 (0.934)	2.497	0.017
	CompP-LingP	0.194 (0.577)	2.023	0.051	0.167 (1.134)	0.882	0.384

**Table 9:** Per-type analysis. Negation

		GPT-4			Llama 3.3		
Negation	ANOVA	Mean (SD)	*F* (2, 70)	*p*	Mean (SD)	*F* (2, 70)	*p*
	DemoP	2.806 (1.546)	3.553	0.034	1.778 (1.333)	4.494	0.015
	LingP	2.333 (1.568)			2.167 (1.320)		
	CompP	3.139 (1.376)			2.500 (1.444)		
	*t*-test	Mean (SD)	*t*-test (df = 35)	*p* (2-tail)	Mean (SD)	*t*-test (df = 35)	*p* (2-tail)
	LingP-DemoP	− 0.472 (1.748)	− 1.621	0.114	0.389 (1.536)	1.519	0.138
	CompP-DemoP	0.333 (1.927)	1.038	0.307	0.722 (1.344)	3.224	0.003
	CompP-LingP	0.806 (1.786)	2.706	0.010	0.333 (1.454)	1.375	0.178

**Table 10:** Per-type analysis. Lexical features

		GPT-4			Llama 3.3		
Lexical features	ANOVA	Mean (SD)	*F* (2, 70)	*p*	Mean (SD)	*F* (2, 70)	*p*
	DemoP	0.028 (0.167)	1.000	0.373	0.000 (0.000)	4.512	0.014
	LingP	0.000 (0.000)			0.111 (0.319)		
	CompP	0.000 (0.000)			0.194 (0.401)		
	*t*-test	Mean (SD)	*t*-test (df = 35)	*p* (2-tail)	Mean (SD)	*t*-test (df = 35)	*p* (2-tail)
	LingP-DemoP	− 0.028 (0.167)	− 1.000	0.324	0.111 (0.319)	2.092	0.044
	CompP-DemoP	− 0.028 (0.167)	− 1.000	0.324	0.194 (0.401)	2.907	0.006
	CompP-LingP	0.000 (0.000)	N/A	N/A	0.083 (0.439)	1.139	0.263

**Table 11:** Per-type analysis. Phonological features

		GPT-4			Llama 3.3		
Phonological features	ANOVA	Mean (SD)	*F* (2, 70)	*p*	Mean (SD)	*F* (2, 70)	*p*
	DemoP	1.889 (1.833)	8.681	< 0.001	6.806 (3.520)	3.097	0.051
	LingP	0.889 (1.214)			5.556 (2.273)		
	CompP	2.389 (1.946)			6.917 (3.557)		
	*t*-test	Mean (SD)	*t*-test (df = 35)	*p* (2-tail)	Mean (SD)	*t*-test (df = 35)	*p* (2-tail)
	LingP-DemoP	− 1.000 (2.330)	− 2.575	0.014	− 1.250 (3.850)	− 1.948	0.059
	CompP-DemoP	0.500 (2.063)	1.454	0.155	0.111 (3.831)	0.174	0.863
	CompP-LingP	1.500 (2.197)	4.096	< 0.001	1.361 (3.217)	2.538	0.016

**Table 12:** Per-type analysis. Out-of-list

		GPT-4			Llama 3.3		
Out-of-list	ANOVA	Mean (SD)	*F* (2, 70)	*p*	Mean (SD)	*F* (2, 70)	*p*
	DemoP	2.417 (1.251)	0.814	0.447	1.778 (0.866)	8.418	< 0.001
	LingP	2.083 (1.795)			1.139 (0.867)		
	CompP	2.000 (1.586)			2.167 (1.384)		
	*t*-test	Mean (SD)	*t*-test (df = 35)	*p* (2-tail)	Mean (SD)	*t*-test (df = 35)	*p* (2-tail)
	LingP-DemoP	− 0.333 (2.111)	− 0.947	0.350	− 0.639 (1.150)	− 3.333	0.002
	CompP-DemoP	− 0.417 (2.005)	− 1.247	0.221	0.389 (1.661)	1.405	0.169
	CompP-LingP	− 0.083 (2.103)	− 0.238	0.813	1.028 (1.682)	3.667	< 0.001

#### Pre-verbal Markers (Table 6)

An ANOVA indicates a significant difference among “DemoP,” “LingP,” and “CompP” for both GPT-4 (*p* = 0.001) and Llama 3.3 (*p* < 0.001). The *t*-tests also show that explicit linguistic cues tend to have a stronger effect than demographic cues alone. Notably, for Llama 3.3, the DemoP condition yields a smaller mean count of pre-verbal markers (0.53) compared to LingP (3.31) and CompP (2.78). This contrasts with GPT-4, where the mean counts are more balanced across DemoP (0.97), LingP (1.44), and CompP (1.89). GPT-4 appears to link this linguistic feature more consistently to the African American demographic, while Llama 3.3 demonstrates a strong capacity to adjust its responses when given explicit AAVE cues, as reflected in the significant jump in pre-verbal markers.

#### Verbal Tense-Number Marking (Table 7)

Only Llama 3.3 shows a significant result in the ANOVA (*p* < 0.001), driven by a clear increase in usage from DemoP (0.14) to LingP (0.78) and CompP (0.64). GPT-4, by contrast, does not show a significant difference (*p* = 0.34), with relatively low and similar mean counts in DemoP (0.19), LingP (0.08), and CompP (0.14). This pattern highlights Llama 3.3’s in-context learning ability: once explicitly instructed, it “learns” and produces more tense-number markers. GPT-4’s usage of this feature stays low, even with direct AAVE cues, suggesting that neither demographic nor linguistic instructions significantly affect its behavior.

#### Nouns and Pronouns (Table 8)

Here, only GPT-4 shows a significant ANOVA result (*p* = 0.010), while Llama 3.3 does not (*p* = 0.083). This appears to be due to GPT-4’s very low mean usage in the DemoP condition (0.03). Overall, GPT-4 rarely produces this feature, with means of 0.03 in DemoP, 0.08 in LingP, and 0.28 in CompP. Meanwhile, Llama 3.3 shows a relatively higher mean (0.47) under DemoP, suggesting it can associate this feature with the African American demographic. Pairwise *t*-tests indicate that the difference between CompP and DemoP is significant for both GPT-4 (*p* = 0.005) and Llama 3.3 (*p* = 0.017), showing that adding explicit linguistic cues helps both models adjust their outputs.

#### Negation (Table 9)

The negation feature stands out because GPT-4 consistently shows higher mean usage across DemoP (2.81), LingP (2.33), and CompP (3.14) than Llama 3.3 (1.78, 2.17, and 2.50, respectively). This contrasts with other features, where Llama 3.3 typically exhibits higher means. For GPT-4, the most notable difference is between LingP and CompP (*p* = 0.010), indicating that adding demographic context on top of AAVE instructions boosts negation usage more than linguistic cues alone. In contrast, Llama 3.3’s main significant difference is between DemoP and CompP (*p* = 0.003), suggesting that combining demographic and explicit linguistic cues leads to more frequent negation than demographic cues alone. This divergence may reflect GPT-4’s stronger tendency to associate negation with the African American demographic.

#### Lexical Features (Table 10)

Both GPT-4 and Llama 3.3 find it difficult to emulate lexical features. GPT-4, in particular, barely uses them, showing mean counts of 0.03 under DemoP and 0.00 under both LingP and CompP (*p* = 0.373 for the ANOVA). While Llama 3.3’s usage also remains low, it does produce a few items when explicitly prompted, with mean counts of 0.00 for DemoP, 0.11 for LingP, and 0.19 for CompP (*p* = 0.014 for the ANOVA). Pairwise tests for Llama 3.3 show that both LingP versus DemoP (*p* = 0.044) and CompP versus DemoP (*p* = 0.006) are significant. This suggests that even if Llama 3.3 does not initially link these features to the African American demographic, it can “learn” and generate them when given explicit AAVE cues.

#### Phonological Features (Table 11)

These results suggest both models carry an internal stereotype about African American speech. Although Llama 3.3 shows higher mean usage across DemoP (6.81), LingP (5.56), and CompP (6.92) than GPT-4 (1.89, 0.89, and 2.39, respectively), both follow the same pattern: CompP > DemoP > LingP. This is unusual for Llama 3.3, which generally produces more features in LingP than in DemoP for most other categories. If we hypothesize DemoP > LingP, a one-tailed test confirms significance for both GPT-4 (*p* = 0.007) and Llama 3.3 (*p* = 0.03)—see Table 7–2 in Appendix 4 for these one-tailed test results. The difference between CompP and LingP is also significant for both models (two-tailed *p* < 0.001 for GPT-4, *p* = 0.016 for Llama 3.3). Thus, simply referring to the patient as African American strongly increases phonological features, indicating a built-in association or stereotype. The fact that both models add phonological markers based solely on referencing “the way many African Americans speak English” underscores this internalized link between demographic cues and specific speech features.

#### Out-of-List (Table 12)

Finally, for the “out-of-list” category, GPT-4 shows roughly the same usage of “unlisted” informal or non-standard English forms across all conditions—whether prompts include demographic cues, explicit AAVE instructions, or both (ANOVA: *p* = 0.447, not significant). In contrast, Llama 3.3 shows a significant rise in these out-of-list features when demographic cues are included (DemoP vs. LingP: *p* = 0.002; LingP vs. CompP: *p* < 0.001). Its “U-shaped” pattern suggests that these additional non-standard English elements increase when the demographic context is mentioned. On the other hand, providing only a list of AAVE instructions (LingP) somewhat reduces these unenumerated features, possibly because the model focuses on the specified prompts and overlooks other variants it “knows.” The fact that demographic information triggers more informal forms indicates that the model “unlocks” a wider range of non-standard English when it detects the demographic cue.

Taken together, these findings show that GPT-4 and Llama 3.3 simulate AAVE in distinct ways. Llama 3.3 tends to produce more features overall when given explicit linguistic details, whereas GPT-4 often relies more on demographic cues, especially for negation and phonological features. In both models, combining demographic and linguistic cues (CompP) yields the highest number of features. Yet neither model perfectly captures the full spectrum of AAVE, and both display what appears to be an internal association (or bias): simply mentioning the African American demographic can trigger more phonological features and “out-of-list” informal English. This pattern suggests the models have pre-existing assumptions linking those linguistic traits to that specific demographic group.

### Out-of-List Features

We examined the “out-of-list” features that each model used more than once to determine whether they align with AAVE. Table 13 presents a condensed summary, showing both the total frequency of these features across all responses and representative example sentences from the models’ outputs.

**Table 13:** Selected “out-of-list” features used more than once by GPT-4 and Llama 3.3, with sample excerpts from the models’ outputs

“Out-of-list” examples	GPT-4	Llama 3.3
Omission of the subject	99	5
	“Just been stayin’ around the house, you know?” (Case2, DemoP)	“Been like that for ‘bout a week now.” (Case5, CompP)
Use of “doc” for “doctor”	55	112
	“Doc, I been feelin’ …” (Case1, DemoP)	“I been havin’ this real bad chest pain, doc.” (Case1, LingP)
Use of “naw” or “nah” for “no”	28	11
	“Naw, doc.” (Case6, CompP)	“So, nah, I don’t think I got no allergies, doc.” (Case3, CompP)
Use of “ya” for “you”	7	10
	“Just got my diabetes, ya know.” (Case3, CompP)	“he was at work, ya know, …” (Case6, DemoP)
Use of “real” for “really”	11	10
	“We been keepin’ it real safe, you know.” (Case4, CompP)	“I been feelin’ real bad, doc.” (Case3, LingP)
Use of “whatnot” for “etc.”	0	9
		“I’ve had my fair share of vaccinations and whatnot, …” (Case1, DemoP)

We acknowledge that these features are not exclusive to AAVE and commonly appear in various informal or regional varieties of American English. For instance, subject omission can occur in casual speech even when not strictly associated with AAVE, and “Naw” or “Nah” for “no” can be found in multiple dialects, including Southern American English. Reducing “doctor” to “doc” is a familiar shorthand used in many informal contexts. Similarly, saying “ya” for “you,” “real” for “really,” and “whatnot” for “etc.” are informal speech habits that appear broadly. Therefore, while such sentences could appear in AAVE, they could also reflect general casual American English usage. To identify speech as definitively AAVE, one would look for consistent patterns and grammatical structures that distinguish it from general American speech, such as the set of AAVE features covered by this study.

Nevertheless, as discussed in Sect. 3.2, the model’s decision to deploy these features under the African American–related cues suggests that LLMs may have internalized probabilistic associations linking certain colloquial expressions with African American speakers. Hence, although these “Out-of-list” forms do not by themselves define AAVE, their increased frequency under demographic cues indicates the models’ inclination to draw on a broader range of informal or dialectal features when simulating an African American voice.

The “out-of-list” category therefore remains open-ended to reflect the ways language models—and real-world speakers—freely draw on informal registers. This underscores the fluidity of dialectal boundaries, while also highlighting how pre-existing associations (or biases) within the model may inadvertently inflate or alter the perceived prevalence of certain features when given demographic cues.

## Discussion

Our study demonstrates that both GPT-4 and Llama 3.3 are capable of using AAVE features across various prompts, with the exception of the BaseP, which served as a benchmark. CompP appears to be the most effective prompt for both models because it contains the most cues. However, for GPT-4, DemoP often yields more AAVE features than LingP, particularly for negation and phonological features, while Llama 3.3 shows the opposite pattern, with LingP > DemoP. Llama 3.3 also generally produces more features overall and shows stronger significant differences across prompts.

One possible explanation for GPT-4’s tendency to produce more features under DemoP than LingP is that GPT-4 seems to have an internal association linking references to an African American demographic with certain AAVE elements, especially phonological variants. Once demographic cues are introduced, GPT-4 “unlocks” a wider range of these features. By contrast, providing an explicit list of AAVE linguistic markers does not activate this internal association as strongly. Meanwhile, Llama 3.3 shows greater sensitivity to explicit linguistic prompts. It readily produces more features when given a list of AAVE cues than when it sees only demographic information, suggesting it relies less on internal stereotypes and more on explicit instructions.

Moreover, different language models, whether from separate providers or updated versions of the same model, naturally exhibit unique behaviors because they vary in training data, parameter tuning, and alignment strategies. This inherent variability explains why GPT-4 and Llama 3.3 do not respond identically, and future updates to either model may further alter their responses. Overall, the difference between these two models underscores that the same prompt strategy—BaseP, DemoP, LingP, and CompP—can produce distinct outcomes depending on how each model encodes and retrieves linguistic and demographic information.

### Observed Biases in Phonological and Out-of-List Features

In the type-by-type analysis, phonological features stood out. Both models, but especially GPT-4, exhibited a strong reliance on demographic cues for triggering phonological variants. This reveals a potential bias: once “African American” is mentioned, these phonological elements appear in greater quantity, even if the prompt does not explicitly request such features. While this can be beneficial for simulating certain dialectal traits, it also raises concerns about stereotyping and over-association of certain language patterns with a specific demographic group.

Moreover, our findings reveal that both GPT-4 and Llama 3.3 generate out-of-list features on their own. These features were not explicitly specified in the prompt, yet they appeared whenever demographic or linguistic cues were provided. Notably, when given only demographic information, the models spontaneously produced a wide range of non-standard English forms. In contrast, when prompts explicitly listed certain linguistic features, the models did not limit themselves to just those items. Instead, they added other elements that may be associated with the African American demographic. This self-driven inclusion of additional dialectal elements can be both an asset (showing more natural dialectal variation) and a liability (risking perpetuation of stereotypes). This emergent behavior opens up new research possibilities. Future studies might explore LLMs’ intrinsic understanding of AAVE through ablation experiments, removing individual features to observe if the model continues to represent them.

### Biases Learned from Pre-training Data

Both GPT-4 and Llama 3.3 were trained on vast text corpora that inevitably reflect language use patterns, including stereotypes and biases, which are prevalent in mainstream or majority-group data. For example, the increased production of phonological features upon encountering the demographic “African American” may stem from an overrepresentation of simplified or stereotypical AAVE examples in certain online sources, while more nuanced or less frequent dialectal forms—such as lexical items like “steady,” “come,” or “finna”—remain underrepresented. This was evident in the models’ tendency to overproduce phonological features and slang associated with African American speech, even when only given the prompt “African American” without specific linguistic cues. Such biases in the pre-training data can cause models to over-associate certain language markers with specific demographics (e.g., viewing all African American speakers as consistently using certain phonological variants). Consequently, LLMs’ reliance on these data distributions can hinder their ability to accurately generalize across the full range of AAVE, especially for less frequent or context-dependent language varieties. The model may appear fluent in some respects but still miss important sociolinguistic nuances.

This not only poses risks of perpetuating narrow or biased portrayals of a dialect group but also diminishes the model’s robustness in real-world interactions, particularly in clinical contexts where communication must be tailored to individual linguistic backgrounds rather than simplified assumptions. For instance, over-reliance on stereotypical language markers could lead to inappropriate or offensive responses in therapeutic or counseling settings, hurting trust between healthcare providers and patients. In scenarios where tailored communication is critical, such as explaining medical procedures or medication instructions, a model’s inability to accurately adapt to diverse linguistic backgrounds could result in confusion or non-compliance. Furthermore, clinicians relying on biased language models might unconsciously adopt narrow views of certain demographic groups, reinforcing systemic inequities in healthcare. These examples highlight the importance of using more detailed and representative training data, as well as thorough and careful evaluation frameworks that ensure language models can handle the full diversity of human language in real-world applications.

One potential way to reduce these biases is to fine-tune or adapt the model using carefully selected datasets that include a wider and more authentic range of AAVE usage. By providing higher quality examples, whether through human annotation or through corpora specifically designed to reflect the diversity of AAVE, developers can guide the model to generate more balanced and representative outputs, not only reducing stereotypical overgeneralizations but also improving accuracy and coverage for less frequent lexical or grammatical features. Such targeted fine-tuning could also use techniques like reinforcement learning from human feedback (RLHF), where cultural and linguistic experts (e.g., sociolinguists specializing in AAVE) collaborate to correct overgeneralizations, reduce stereotypical outputs, and encourage the model to reflect the true diversity of AAVE. For example, experts could identify and remove biased or overly simplified examples from the training data, while adding more context-rich and varied instances of AAVE usage. This can help LLMs capture a fuller spectrum of AAVE usage and reduce the reliance on a narrow set of features commonly found in mainstream data.

Interestingly, our empirical findings with Llama 3.3, a more recently developed model than GPT-4, offer some evidence that more recent or better-refined models may already be bridging these gaps. Compared to GPT-4, Llama 3.3 not only generated a greater overall number of AAVE features across both demographic and linguistic cue prompts, but also more closely reflected the pattern CompP > LingP > DemoP. This suggests that as LLMs incorporate more up-to-date, refined datasets and apply advanced alignment strategies, they can better capture a wider range of dialectal features and nuances. In other words, improvements to underlying model may gradually mitigate the limitations highlighted in earlier-generation models.

Additionally, ongoing research into prompt engineering, including our own, can help refine the instructions given to LLMs, making them less likely to rely on pre-trained stereotypes. For example, carefully structured prompts that include nuance about code-switching, regional AAVE variants, or more context-specific cues may help reduce the model’s inclination to apply one-size-fits-all phonological features whenever it sees the demographic cue “African American.” This method has shown promise in a study [32], which tested five LLMs across 107 countries and territories using data from the World Values Survey. By adjusting prompts to include explicit cultural or demographic information, the study found that the models, which initially reflected Western, English-speaking norms, could be guided to better align with local cultural values in 71–81% of cases. This suggests that similar techniques, like using explicit AAVE cues or cultural prompting, could help reduce biases learned during pre-training, whether they relate to nationality, regional dialect, or minority language forms.

### Absent Features

Another key finding is that certain features, such as question forms and existential-locative constructions, did not appear in either model. While various factors may explain this absence, it does not necessarily mean the models are incapable of producing them. For instance, although our main experiment showed no instances of question forms, when we explicitly prompted GPT-4 using LingP with “Do you have any questions?”, we observed the response, “Well, doc, what you think this chest pain be about?”, which demonstrates the use of AAVE question forms. This suggests that the absence of these features in the main experiment may reflect the conversational context rather than an inherent inability of the models to generate them.

The inherent structure of a doctor-patient interview, with the doctor typically asking questions and the patient responding, naturally favors certain verb forms, negation, and phonological markers, while minimizing the chance for existential/locative statements or question inversion. Existential-locative forms in AAVE (e.g., “It’s a dog in here,” “They got some hungry women here,” “Here go my keys”) allow speakers to express the presence or location of someone or something without using Standard English “there is/are.” Typically, these forms arise in statements about where something is located. In doctor-patient exchanges, the patient generally reports symptoms, expresses concerns, or provides personal/family medical history. These contexts rarely demand explicit statements of existence or location.

Questions in AAVE can appear without the typical subject-auxiliary inversion of Standard English (e.g., “Where that is?” instead of “Where is that?”). Embedded questions may also retain an inversion that Standard English does not (e.g., “I asked her could I go with her?”). In these clinical dialogues, the physician is more likely to ask the questions. The patient typically answers or explains, which puts them in a declarative rather than interrogative mode. As a result, the patient seldom needs to pose questions, so question forms do not arise naturally.

Given how context-sensitive dialect features are, simply mentioning “African American” demographics or listing AAVE elements does not guarantee that all possible linguistic patterns will appear. Even if the model “knows” these forms, LLM outputs are heavily shaped by context. If the scenario rarely calls for mentioning existence or location, or for posing a question, the model’s internal weighting for these constructions remains low. Therefore, without an explicit prompt (e.g., “Tell me about what’s around you”, “Do you have any questions?”), these features tend to remain dormant.

### Limitation

Despite the promising findings of this study, several limitations must be acknowledged. First, the focus was largely on text-based simulations of linguistic traits, without considering broader demographic variables such as gender, age, geographical location, or socio-economic status. This narrow scope may limit the generalizability of our findings, as language use often intersects with multiple social factors. Future work should address this gap by expanding experimental designs to include a wider variety of demographic and sociolect variables.

Second, since our study only addressed written aspects of AAVE through text-based LLM outputs, important phonological elements of spoken communication—such as intonation, rhythm, and accent—were excluded. This omission is especially relevant for dialects like AAVE, where pronunciation and accent play a critical role in shaping meaning and identity. To address this gap, future research could explore the use of multimodal LLMs or audio-based models capable of understanding and simulating speech patterns. As noted in prior work [16], it is crucial for voice-based technologies in both healthcare and everyday contexts to accurately recognize regional dialects and cultural linguistic patterns. This ensures that users can communicate in their own linguistic style without the need to code-switch, making digital tools more inclusive and accessible.

### Application

The ability of LLMs to simulate AAVE features presents opportunities to address systemic inequities in healthcare communication. However, these tools must be designed with intentionality, transparency, and collaboration with cultural stakeholders to avoid perpetuating harm. Below, we outline actionable applications and guidelines to ethically integrate LLMs into clinical practice, education, and research.

#### Potential Applications

##### Cultural Competency Training

LLM-based patient simulations can be developed to enhance cultural competency training for healthcare professionals. In [33], the authors developed virtual patient simulators using a Siamese LSTM architecture, which improved medical students’ diagnostic reasoning and learning outcomes through interactive feedback and targeted review suggestions. Meanwhile, [34] shows that immersive simulations through virtual reality (VR) technology can train empathy. Building on these findings, clinicians and medical students can practice empathetic, culturally sensitive communication by interacting with simulated patients speaking a variety of linguistic styles, powered by LLMs. Specifically, such AI-driven patient modules can replace or supplement traditional scripted approaches, such as the USMLE’s CCS scenarios, dynamically exposing learners to diverse linguistic styles while helping them recognize and address biases or communication barriers.

##### Patient-facing Tools

We can design telehealth or appointment-scheduling chatbots that “code-switch” to reflect patient dialect choices. This approach can enhance patient comfort, trust, and engagement. Similarly, culturally attuned health education tools could present patient education content in more accessible ways, ensuring materials resonate with specific patient communities. However, these chatbots require careful oversight to reflect linguistic variations like AAVE without reinforcing harmful stereotypes. To mitigate this, pilot-testing with community stakeholders, such as AAVE speakers, sociolinguists, and healthcare providers, is essential. Iterative feedback loops can help refine outputs to balance authenticity and clinical appropriateness.

##### Clinical Documentation

LLMs also have potential benefits for medical transcription and documentation. Tools that sensitively accommodate demographic-specific linguistic traits in clinical conversations can create context-aware summaries, maintaining the authenticity of speech patterns while translating them into clinically useful notes. This approach can reduce miscommunication due to dialectal differences.

##### Dataset Development

LLMs can be used to generate synthetic data featuring linguistically diverse texts for underrepresented dialects, filling gaps in existing datasets and enabling more comprehensive studies of linguistic diversity. For example, a recent study [35] introduced a multi-agent LLM system that transforms English clinical notes (from the ACI-Benchmark dataset) into culturally appropriate Arabic medical dialogues, incorporating the Saudi Najdi dialect to address the lack of publicly available Arabic medical conversation datasets.

##### Multimodal and Audio-based Approaches

While our study focused on text-based outputs, there is significant potential for multimodal models that capture key phonological aspects of dialects like AAVE (e.g., accent, intonation, rhythm). Such audio-enabled systems could benefit any of the applications above. For instance, a medical student might interact with an AI-driven “virtual patient” that uses both text and speech in AAVE, while a code-switching chatbot could not only write but also speak with natural AAVE phonological markers. Researchers could generate large-scale audio datasets using text-to-speech or speech-recognition modules (e.g., Tortoise TTS, Whisper, wav2vec) integrated with LLM-driven dialogue, producing synthetic audio clips that reflect authentic pronunciation. To ensure respectful reproduction of spoken dialect, these applications require careful evaluation methods, including systematic phonetic analysis and listening tests with native speakers or linguists to assess authenticity.

#### Guidelines for Implementation

To ensure LLMs are deployed ethically and responsibly, we propose the following guidelines. These principles extend beyond AAVE to any language or dialect that technology seeks to represent, and they rely on collaboration with the very communities being portrayed.

##### Involving Experts

First, it is necessary to involve cultural and clinical experts at every stage. Biases or overgeneralization can be mitigated through regular audits for stereotypical or insensitive language by cultural experts, such as sociolinguists, healthcare professionals, and community members. For instance, if an LLM-based patient simulator defaults to slang in a way that stereotypes rather than authentically reflects communication, cultural experts can flag this for revision. Using automated bias detection tools, such as IBM’s AI Fairness 360 [36], can also be effective but it is mandatory to involve human annotators with cultural expertise to catch subtle cues.

##### Thoughtful Prompt Engineering

Prompt engineering should prioritize context over demographics. Rather than relying on generic, monolithic demographic triggers like “African American,” prompts should incorporate situational and individual variables to encourage more nuanced linguistic generation and reduce stereotyping. For example, a well-designed prompt might specify: “Generate a dialogue where a 60-year-old female patient from Chicago, who code-switches between AAVE and formal English, discusses diabetes management with a clinician.” This approach acknowledges dialect fluidity and avoids conflating language patterns with assumptions about socio-economic status or health behaviors. A/B testing different prompts with cultural experts can further refine outputs, comparing responses to vague versus detailed prompts and scoring them for authenticity and clinical appropriateness.

##### Data Transparency

When using LLM-generated data for training clinicians, any real patient data must be fully de-identified and meet HIPAA (for U.S. patients), or other relevant data-protection standards. Anyone using an AI system for patient-facing tools should be transparently told that an AI (rather than a human) is generating or interpreting their conversation and should also have easy opt-out options. Also, the patient should understand how their data (such as chat transcripts or personal information) will be collected, stored, and potentially used (for example, to improve the AI model). This ensures patients can make an informed decision about whether they are comfortable participating in these AI-assisted interactions.

## Conclusions

This research investigates whether LLMs, specifically GPT-4 and Llama 3.3, can replicate AAVE in simulated clinical dialogues in ways that might lessen communication barriers between patients and physicians. The results of the simulation illustrated that including demographic or explicit AAVE markers led the models to generate AAVE features. Combining demographic and linguistic cues triggered the highest counts of these features. However, they do not capture its full diversity and tend to miss some grammatical forms when the conversational context does not elicit them. Differences between the two models also emerged, with GPT-4 appearing more sensitive to demographic directive and Llama 3.3 responding strongly to explicit lists of linguistic features.

Both models’ tendency to generate extra phonological and out-of-list features when provided with demographic cue underscores their reliance on internalized distributions of training data, which may include biased or limited examples of AAVE, reflecting only certain patterns deemed “representative” by popular media or internet sources. These narrow depictions can reinforce harmful assumptions about Black speech, linking it solely with informal slang or superficial phonological features, while disregarding the dialect’s nuanced grammar, historical roots, and regional variations. This highlights both the promise of LLM-driven simulations for improving cultural competency and the dangers of unintentionally reinforcing stereotypes. By combining expert input with careful prompts and transparent practices, LLMs can move away from simplified stereotypes and offer more respectful and accurate simulations of AAVE in clinical or educational settings.

Ultimately, this work is an important step towards using AI to address linguistic inequities in healthcare. While it focused on whether LLMs can simulate AAVE, the results show the broader potential of these models to support more inclusive patient-provider communication if they are built with strong ethical standards and shaped by community input. The next step is to turn this potential into real-world applications, such as AI-driven cultural competency modules for medical education or dialect-aware chatbots for telehealth platforms, to better address underrepresented dialects. Future research could also expand to other dialects and languages, ensuring that AI tools capture the full range of human language use. Work on multimodal systems may help capture the rich phonological features of speech, and longitudinal studies could measure how these tools affect patient outcomes in clinical settings. By focusing on transparency, ongoing input from the community, and training practices that limit bias, developers can build models that celebrate linguistic diversity rather than reinforce stereotypes. In doing so, AI can help bridge communication gaps and advance health equity by respecting the voices of marginalized communities.

## Supplementary Information

Below is the link to the electronic supplementary material.

Supplementary file1 (PDF 887 KB)

Supplementary file2 (PDF 202 KB)

Supplementary file3 (PDF 116 KB)

Supplementary file4 (PDF 162 KB)

Supplementary file5 (ZIP 75 KB)

## Data Availability

No datasets were generated or analysed during the current study.

## Abbreviations

*AAVE*: African American Vernacular English
*AI*: Artificial Intelligence
*ANOVA*: Analysis of variance
*BaseP*: Baseline Prompt
*CCS*: Computer-Based Case Simulations
*CompP*: Comprehensive Prompt
*DemoP*: Demographic Prompt
*IAA*: Inter-annotator Agreement
*LLMs*: Large language models
*LingP*: Linguistic Prompt
*USMLE*: United States Medical Licensing Examination
