# Supplementary material for: Enhancing Patient-Physician Communication: Simulating African American Vernacular English in Medical Diagnostics with Large Language Models
Source: J Healthc Inform Res. 2025 Mar 11;9(2):119–53. doi: 10.1007/s41666-025-00194-9 (PMC12037967; doi:10.1007/s41666-025-00194-9)
Supplement: Supplementary file 1 — Supplementary file1 (PDF 887 KB) [file 41666_2025_194_MOESM1_ESM.pdf]

Appendix 1

Annotation Guidelines

1. Types

Pre-verbal markers

- Omission of "is" and "are"
- Invariant “be”
    - Habitual actions
    - Contractions of "will/would be"
- “been”/“bin”
    - Unstressed "been" for present perfect
    - Stressed "been" for the action that happened a long time ago
- Use of “done” for a distant past tense
- Use of “be done” for a future perfect tense
- Use of “had” for a past tense
- Use of double modals

Verbal tense-number marking

- Absence of third person singular present -s, doesn't, or has
- Use of "is" and "was" for plural and second person subjects
- Use of past tense for past participle
- Use of past participle for past tense
- Use of verb stem (root forms) for past tense
- Reduplicated tense marking

Nouns and pronouns

- Unmarked possessives
- Unmarked plural forms
- Regularization of irregular plural nouns
- Use of "an 'em", "and 'em", "nem" to mark associative plurals
- Appositive or pleonastic pronouns
- Use of “y'all” for the 2nd person plural
- Use of demonstrative “them”
- Omission of relative pronoun

Negation

- Negative concord
- Negative inversion

- Use of "ain't" as a general preverbal negator
- Use of "ain't" + "but", and "don't" + "but" to indicate "only"

Questions

- Formation of direct questions without inversion
- Inversion in embedded questions

Existential-locative construction

- Use of existential "it", "they" (or "dey")
- Use of existential "they got"
- Use of "here go" as a static locative or presentational form

Lexical Features

- Use of "steady" for consistent, persistent, or repeated action
- Use of "come" to imply the speaker's indignation
- Use of "finna" to indicate immediate future actions

Phonological Features

- Replacement of final "ing" with "in'"
- Omission of unstressed syllables at the beginning and middle

Out-of-list

Any deviations from 'standard English' not covered by the above list

---

2. How to Define Boundaries

The primary rule is to tag each word or phrase that exhibits linguistic features.

If a feature involves omitting a word, mark both the immediately preceding and following words. However, if the omitted word would have been the first word in the sentence (such as the subject), only mark the following word, since there is no preceding word.

This process often results in overlaps.

Examples

Below are examples illustrating how to define boundaries in cases that might be ambiguous.

Pre-verbal markers

When “is” or “are” are omitted, mark from the subject up to the following word.

- **He the** one
- **They all grown** up now

- **It getting** worse
- **He a** healthy boy

If “been” immediately follows “ain’t” (e.g., “I ain’t been ...”), do not annotate “been” under Pre-verbal markers. In this context, “ain’t” is a general preverbal negator and it’s unclear which verb form “ain’t” is replacing.

- We ain't been nowhere out of the ordinary

Nouns and pronouns

When appositive or pleonastic pronouns follow a noun, mark both the noun being explained and the explanatory pronoun. However, rather than marking the entire noun phrase, only mark the main noun.

- My bridge **partners, they** all be lookin’ good too
- My **kids, they** come visit me
- This **pain, it** don’t get worse

When a relative pronoun is omitted, identify its antecedent. Mark both the antecedent and the word immediately following. However, if the omitted pronoun would have appeared at the start of the sentence—or if the antecedent is the entire sentence or too difficult to determine—mark only the following word.

- He the **one brought** me in
- She works in advertising sales, **keeps** her pretty busy

Negation

If “ain’t” (a general preverbal negator) appears in combination with other negation features, do not annotate every occurrence of “ain’t” as a separate feature.

In the case of negative concord, the text starting from the first negator up to and including the last negator in the sentence should be marked.

- I **ain’t been nowhere** recently
- I was 27 and 32. **Ain't had no major illnesses or nothin'** in the past 12 months
- I **don't take no over-the-counter stuff or no herbal supplements, neither.**
- **Ain’t nobody** around me been sick

When inversion happens due to negation, mark from the auxiliary verb that includes negation to the end of the subject phrase.

- **Ain’t nobody around me** been sick
- **Ain't nothin' out of the ordinary** been goin' on

Note that the sentence "Ain’t nobody around me been sick" and "Ain't nothin' out of the ordinary been goin' on" should be annotated twice:

1. For **negative concord**
2. For **inversion due to negation**

This indicates that the sentence exhibits two distinct elements of AAVE related to negation, both falling under the "Negation" label type. Naturally, this results in overlaps for most parts of the annotations.

When it’s confusing whether to annotate once or multiple times—especially if the same label type applies—consider whether multiple distinct features are truly present.

For example, the sentence “Ain’t nobody around me been sick” could have been expressed as “Nobody around me ain’t been sick.” In that hypothetical case, there would only be one negation feature to annotate. However, the actual sentence not only includes negative concord but also inversion triggered by negation, resulting in two separate annotation instances. Even though both fall under “negation,” these are two distinct features, and each must be annotated.

Note that "Ain’t" is not separately annotated.

Phonological features

- **Havin’** this real bad pain in my chest
- I ain't left the city in months, **'cept** for my regular walks and bridge tournaments
- Ain't nobody **'round** us been sick lately
- It started **'bout** 45 minutes ago, **outta** nowhere

Out-of-list

The omission of certain elements of a sentence is common—mark the preceding and following words as outlined by the primary rule (or only the following word if it’s at the start of the sentence)

- **But ain’t** nothing major in the last year, no
- **Not** breathing easy
- **Been** healthy

Any reduced (contracted) forms of 'standard English' pronoun that are not explicitly listed under a specific feature would be included here.

- My regular doctor prescribed **'em** for me

Informal or colloquial expressions not unique to AAVE, but appearing within informal speech, should also be marked under this category.

- He been wheezin' and coughin' **somethin' awful**

---

3. Annotation tool

Label Studio https://labelstud.io/guide/labeling

1. Select the label that you want to apply to the region.

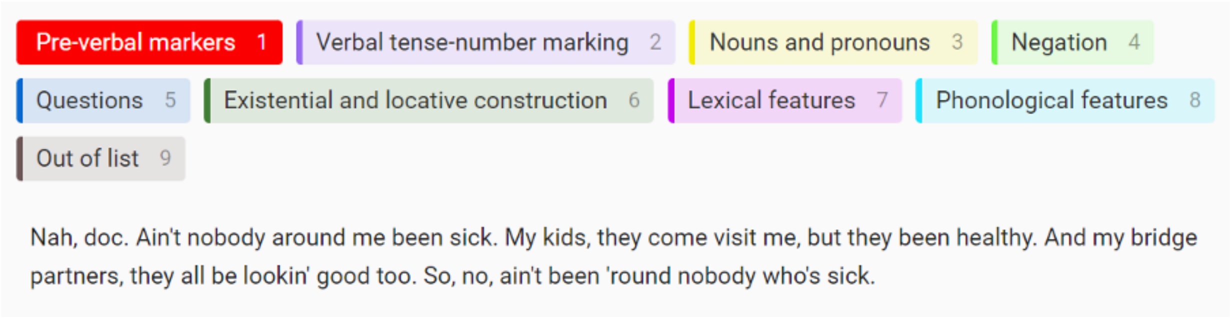

2. Draw the bounding box or highlight the text that you want to label.

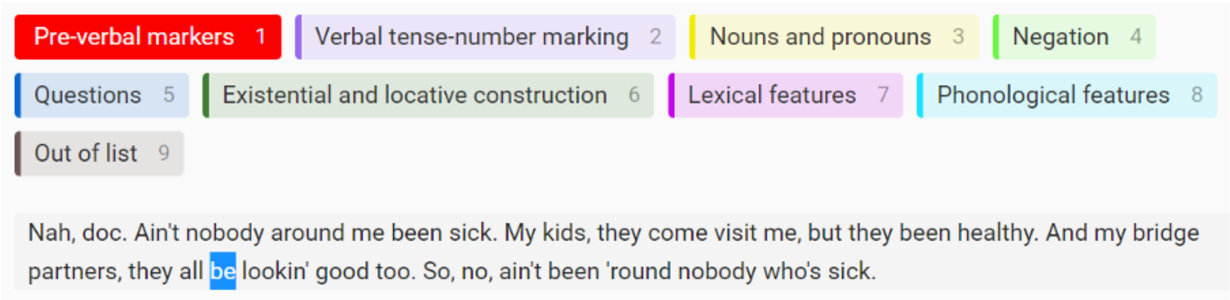

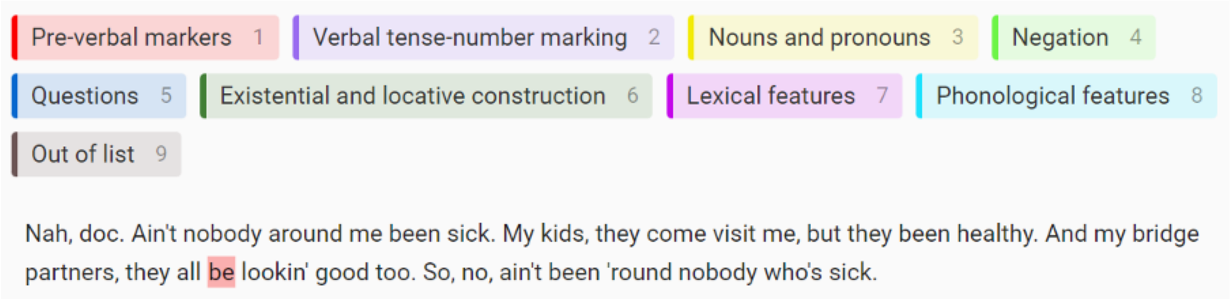

3. Continue labeling regions until you've completed annotating the task.

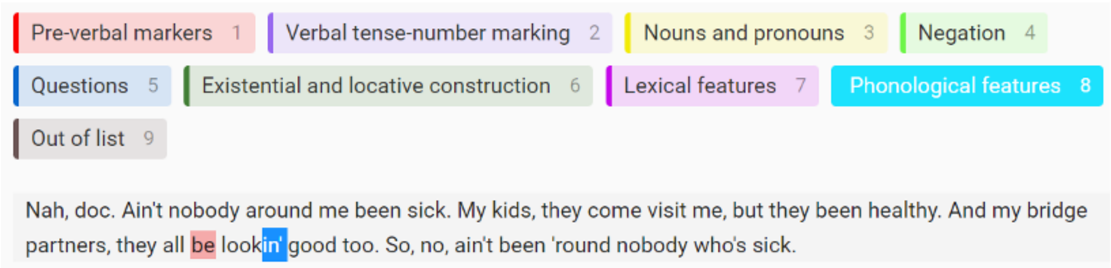

4. Click **Submit** to save the completed annotation and move on to the next task.

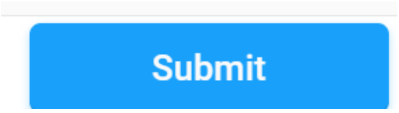

---

4. Annotation Examples

Pre-verbal markers

No, I ain't had none of them. I been goin' to my prenatal appointments regular, and they all been sayin' my pregnancy been goin' good. I had a check-up two weeks ago, and everythin' was fine. I been takin' my prenatal vitamins like I'm supposed to, and I ain't had no problems till these headaches started. My mom, she had endometrial cancer a couple years ago, but I ain't had nothin' like that.

Verbal tense-number marking

Coworker: Yeah, doc, he be takin' a bunch of medications for his emphysema and asthma. He got an inhaler, and he take some pills too. Let me see... (rummages through patient's bag) He got his albuterol inhaler, and he take some prednisone, and... (reads from a piece of paper) ...some spiriva and symbicort. He also take some over-the-counter stuff like acetaminophen for pain, and he been takin' some vitamin D supplements too. His doctor told him to take 'em, so he been takin' 'em regular. (to the patient) You ain't forgettin' to take none of your meds, was you? (patient moans in response) Coworker: Sorry, doc, like I said, he can't talk right now. But I can give you the names of all his medications and the doses if you need 'em. I got the list right here. (pulls out a piece of paper) His wife, she be the one who usually keep track of all his meds, but she ain't here right now. She told me what he been takin', though.

Nouns and pronouns

He be takin' his inhalers for his asthma, and he been takin' some medication for his emphysema, doc. I think it's them steroids, but I ain't exactly sure, his wife be the one who keep track of all that. He also be takin' some over-the-counter stuff for his allergies, but I don't know what kind, it's them pills he get from the store. He ain't never mentioned no herbal supplements, so I don't think he be takin' none of them. He had them medications with him at work, but I didn't bring 'em with me to the hospital, they still back at the truckin' company.

Negation

I ain't got no allergies, doc. None that I know of, anyway. I can eat whatever I want, take whatever meds I need, and I don't never have no reactions or nothin'. My doctor, he always ask me about allergies, and I always tell him the same thing: I ain't got none. So, you don't have to worry 'bout me bein' allergic to nothin', 'kay?

Lexical Features

I been havin' this real bad chest pain, doc. It started 'bout 45 minutes ago, and it's been steady hurtin' me somethin' awful. It's on my left side, and it radiate to my jaw and back. I'd say it's an 8 out of 10, pain-wise. I'm gettin' a little short of breath, too, but it ain't gettin' no worse when I breathe or move around. I just feel kinda nauseated, that's all. Ain't no fever, cough, or nothin' like that, though.

Phonological Features

I'm havin' some bad chest pain, doc. It started 'bout 45 minutes ago, and it's been hurtin' somethin' awful. It's on my left side, and it's sharp, like somebody's stabbin' me. It's also goin' up to my jaw and down my back. I'd say it's an 8 out of 10, pain-wise. I'm gettin' a little short of breath too, but it ain't gettin' no worse when I breathe or move around. I just feel kinda nauseous, that's all.

Out-of-list

(coworker speaking on behalf of the patient) Ahh, exposed to someone sick? (thinks) Now that you mention it, doc, his wife was feelin' a little under the weather last week. She had a cold or somethin', but nothin' too serious. And one of the guys he works with, his name's Joe, he's been out sick for a few days with the flu. (nods) Yeah, that's right. My man here was just talkin' about how Joe was out and how they was short-staffed. But I don't think he was around Joe too much, 'cause Joe's been out for a bit. (pauses) And then there's the truck stop, doc. He stops at them truck stops all the time, and you never know who's been in and out of there. (shrugs) He could've picked up somethin' from someone there, but I don't know for sure. (looks at the patient) He's always been careful, though, washin' his hands and whatnot. (sighs) I just don't know, doc. This is all just so sudden.
