# Supplementary material for: Enhancing Patient-Physician Communication: Simulating African American Vernacular English in Medical Diagnostics with Large Language Models
Source: J Healthc Inform Res. 2025 Mar 11;9(2):119–53. doi: 10.1007/s41666-025-00194-9 (PMC12037967; doi:10.1007/s41666-025-00194-9)
Supplement: Supplementary file 2 — Supplementary file2 (PDF 202 KB) [file 41666_2025_194_MOESM2_ESM.pdf]

Appendix 2

Linguistic Features of African American Vernacular English

1. Grammatical Features

A. Pre-verbal Markers

Omission of “is” and “are”

In Standard English, verbs such as "is" and "are", indicating present tense states and actions, are often contracted, whereas in African American Vernacular English (AAVE), they are omitted[7, 11, 14]. This omission especially often occurs before phrases, progressive verbs, adjectives, and negatives[1].

"She funny"[4]

"She nice"[13]

"They acting silly"[13]

Invariant “be”

I. Habitual actions

Researchers have consistently found that this use of the invariant "be" (sometimes rendered as "bees") is indicative of a habitual aspect[4]. It requires the support of the auxiliary verb "do" in questions, negatives, and tag questions. This habitual usage is distinct from phonological variations seen with contractions of "will" and "would"[13]

"He be walkin'"[11]

"He be talking all the time"[4]

"She don't be sick, do she?"[11]

"Do he be walking everyday?"[11]

II. Contraction of "will/would be"

The second type of "Invariant BE" relates to the future tense "will be" or "would be". This is essentially a byproduct of the phonological rule that deletes the contracted "'ll" from "will", or "'d" from "would"[11, 13].

"He be here tomorrow"[11]

"When June come, I be outta school and outta work"[6, 8]

“been”/”bin”

In African American Vernacular English (AAVE), the use of "been" has two notable forms that differ in terms of stress and meaning.

I. Unstressed "been" for present perfect

This is used in a manner similar to Standard English's "has/have been" in the present perfect tense. Unlike its stressed counterpart, the unstressed "been" can be paired with time adverbials like "since last week" and does not indicate remoteness[11].

"He been sick"[11]

"They been called the cops, and they're still not here"[3, 8]

II. Stressed "been" for the action that happened a long time ago

This indicates that an action or state began a considerable time ago and is used before a verb in its past tense form[13]. For instance, in the sentence "She been married", "been" highlights that she became married a long time ago. different interpretations may arise when the verb following "been" can be interpreted as either a past tense or a participle. This similarity, known to be "camouflage" in linguistics, can lead to misunderstandings between speakers of different linguistic backgrounds. Some non-AAVE speakers might interpret it similar to "has been" (omitting the 's'). For example, when asked based on the previous sentence, "Is she still married?", many white speakers might expect the answer to be "No," interpreting "has been" without a duration modifier as suggesting completion. Conversely, many Black speakers would more likely respond with "Yes," seeing the continuous aspect represented by "been" in AAVE[8].

"She bin married"[11]

"He bin ate it"[11]

Use of “done” for a distant past tense

It indicates actions that have been completed in the past. It often denotes a distant past tense[4], but as highlighted by Mufwene[8], it can be used to reference actions completed in the recent past. "DONE" is positioned as a preverbal auxiliary accompanying past tense verb forms[13]. Additionally, "done" can co-occur with "been" in both "done been" and "been done" formations[11].

"He done failed out"[4]

"It don't make no difference, 'cause they done used all the good ones by now"[8]

"I done told you not to mess up"[13]

"She done did it"[13]

Use of “be done” for a future perfect tense

It is used in a manner akin to the future perfect tense in Standard English, such as 'will have done'. It denotes both the completion of an action and its anticipated occurrence in the future[13]. This tense typically comes before the first of two projected actions, emphasizing that the initial action will be finalized prior to the onset of the subsequent one. Illustrative examples include: "My ice cream's gonna be done melted by the time we get there" and "So they can be done ate their lunch by the time they get there"[8]. Additionally, Dayton proposes an alternative interpretation for this linguistic form. It insinuates an inevitable consequence stemming either from a general condition or a specific event[6]. This interpretation is particularly prevalent in threats or warnings, as observed in expressions like: "If you love your enemy, they be done eat you alive in this society" and "I'll be done killed that individual if he tries to lay a hand on my child again"[13].

"My ice cream's gonna be done melted by the time we get there"[8]

"We be done washed all the cars by the time Jo gets back with the cigarettes"[2, 8]

"She be done had her baby"[13]

Use of “had” for a past tense

'had' is employed to mark the simple past[4, 11]. It can be paired with either past or perfect verb forms to denote past events[13].

"Then we had went outside"[11]

"What had happened was"[4]

Use of double modals

The occurrence of double modals is possible[4]. While some double modals, like "might could,"(equivalent to "might be able to" in SE) are shared with southern white vernaculars, others like "must don't" (equivalent to "must not" in SE) are more uniquely associated with AAVE[11].

"I might could have done that"[4]

B. Verbal Tense-Number Marking

Absence of third person singular present -s, doesn't, or has

The ending -s is often omitted in the third person singular present tense verb[4]. Also, instead of using "doesn't" or "has," AAVE often employs "don’t" and "have" respectively[11, 13].

"He talk too much"[4]

"She walk"[13]

"She have money"[13]

"She run everyday"[13]

Use of "is" and "was" for plural and second person subjects

"is" can replace "are" or "am," and "was" can be applied universally to all past tense forms of the verb "BE." It's noteworthy that the leveling or generalization of the past tense "BE" as "was" is more prevalent than that of the present tense[13].

"The folks is home"[13]

"y'all is here"[13]

"They wasn't there"[4]

"The folks was there"[13]

"y'all was here"[13]

"They is some crazy folks"[11]

"We was there"[11]

Use of past tense for past participle

Past tense verbs can function as participles[8, 11, 13].

"I had went down there"[13]

Use of past participle for past tense

Participles are employed where standard usage would use the simple past tense or preterite form (V-ed)[8, 13].

"They seen it"[13]

Use of Verb Stem (Root Forms) for Past Tense

The verb stem, also referred to as the root or base form, is used in place of the simple past tense or preterite form (V-ed)[13].

"They run there yesterday"[13]

Reduplicated Tense Marking

Often termed as "reduplicated tense marking" or "double tense marking", this feature involves the regularization of irregular past-tense verbs in standard English. Specifically, regular past-tense verb endings, typically "-ed", are appended to verbs that are conventionally irregular, leading to their "double tense marking"[13].

"I seened her"[4]

"Everybody knowed him"[13]

C. Nouns and Pronouns

Unmarked possessives

In the possessive noun form, the -(')s ending can sometimes be absent, as seen in phrases like 'The dog tail was wagging' and 'The man hat was old'. However, the absolute possessive pronoun might be regularized as 'mines' or 'hers'[4, 11, 13].

"John house"[11]

"My mama house"[4]

"The dog tail was wagging"[13]

"The man hat was old"[13]

"The book is mines"[11]

Unmarked plural forms

Measure nouns in AAVE frequently lack the plural -s, exemplified in phrases like "I got 50 cent" or "It's four mile from here." Although this absence of the plural -s can be observed in various English varieties, AAVE extends this feature in instances like "some dog" or "two boy", though such occurrences are not widespread[13].

"Fifty cent"[4]

"It's four mile from here"[13]

Regularization of irregular plural nouns

Irregular plurals, like 'deer,' 'fish,' and 'sheep,' become regularized. Examples include "oxes" instead of "oxen" and "gooses" rather than "geese." This also extends to phrases like "three sheeps" and "two corns." In some cases, you might even find redundant marking like "two firemens" or "childrens"[8, 13].

"Oxes", "Gooses"[13]

"Two firemens", "Childrens"[13]

"Three sheeps", "Two corns"[11]

Use of "an 'em", "and 'em", "nem" to mark associative plurals

The associative plural "an 'em" is used to denote a person and their associates. it's a shorthand way of saying "and them" or "and the others." For instance, "Jerome an 'em" translates to "Jerome and his friends"[11, 13].

"Jerome an 'em"[13]

"Derek an' em will be there"[13]

Appositive or pleonastic pronouns

An appositive or pleonastic pronoun emphasizes or restates a noun for emphasis, clarity, or syntactic reasons, as in "That teacher, she yell at the kids," where "she" reinforces "That teacher"[11].

Use of “y'all” for the 2nd person plural

The term "y'all," a contraction of "you all," is prevalently used as the 2nd person plural form of "you"[4, 8, 10, 13].

"y'all done now"[13]

Use of demonstrative “them”

The object form "them" is used in place of a demonstrative adjective "those" or "these". For instance, one might say "She likes them apples" as opposed to the conventional "She likes those apples"[13].

"She likes them apples"[13]

"I love them shoes"[13]

Omission of relative pronoun

relative pronouns such as "who," "which," "what," and "that" are often omitted in embedded sentences. For example, instead of saying "It's a man who came over here talking trash," one might hear "It's a man come over here talking trash"[13]

"You the one she knows"[4]

"It's a man took it"[13]

"That's the man come here"[11]

D. Negation

Negative concord

negation is indicated more than once in a single expression or clause. This involves negating both the auxiliary verb and all indefinite pronouns in a sentence. In Standard English, the use of two negatives typically renders a statement positive. However, in African American Vernacular English (AAVE), employing multiple negatives serves to intensify the negative meaning[10, 13].

“He don't see nothing”[4]

“She didn't do nothing”[13]

“I ain't no ugly dude”[10]

Negative inversion

A negative auxiliary is placed at the beginning of the sentence, often followed by an indefinite subject. Such inversions serve to emphasize the statement, particularly when stress is placed on the indefinite subject. However, it's important to note that this inversion doesn't occur when the subject is a proper noun, preceded by 'the', or accompanied by a possessive article[13]. For instance, while "Don't nobody like him" and "Ain't nobody gonna find out" are valid examples of negative inversion, "Ain't Mary gonna find out" and "Ain't the teacher gonna find out" are not[10].

"Don't nobody like him"[13]

"Ain't nobody home"[13]

"Ain't nobody gonna find out"[13]

Use of "ain't" as a general preverbal negator

"ain't" can represent a variety of Standard English negations including "haven't," "hasn't," "am not," "isn't," "aren't," and even "didn't"[5, 9, 13].

"He ain't shy"[4]

"I ain't go yesterday"[13]

Use of "ain't" + "but", and "don't" + "but" to indicate "only"

"ain't" or "don't" can be used in conjunction with "but" to convey the meaning of "only" or "no more than"[11].

"He ain't but fourteen years old"[11]

"They didn't take but three dollars"[11]

E. Questions

Formation of direct questions without inversion

Direct questions can be formed without inverting the subject and the auxiliary verb. This absence of inversion, usually accompanied by a rising intonation, can be typically observed in questions such as "Where that is?" or "Why I can't go?" Such structures are especially prevalent in wh- questions and syntactically simple sentences[13].

"He is behind me?"[4]

"Where that is?"[13]

"Why I can't go?"[13]

Inversion in embedded questions

While most embedded questions in Standard English would typically use "if" or "whether" without inverting the subject and the auxiliary verb, AAVE might retain this inversion. For example, instead of saying "I asked her if I could go with her," one might say, "I asked her could I go with her"[13].

"I asked her could I go with her"[13]

F. Existential-Locative Construction

Use of existential "it", "they" (or "dey")

"it" and "they" can denote the existence or presence of something or someone. It resembles the function of the word "there" in Standard American English (SAE) in constructions like "there is" or "there are."

"It's a dog in here"[4]

"It's a J Street in DC"[13]

"They's a J Street in DC"[13]

"It's a school up there"[11]

Use of existential "they got"

"they got" can serve as a plural equivalent to the singular "it is" in AAVE, offering another alternative to "there are"[11].

"They got some hungry women here"[11]

Use of "here go" as a static locative or presentational form

"here go" functions in a manner similar to Standard English's "here is" or "here are"[11].

"Here go my own"[11]

2. Lexical Features

Use of "steady" for consistent, persistent or repeated action

"steady" is used to indicate habitual or intensified actions. This continuative intensifying activity suggests actions that are persistent or consistently done. It often comes between invariant habitual be and a progressive verb[13].

"He steady talking"[4]

"Ricky Bell be steady steppin in them number nines"[11]

Use of "come" to imply the speaker's indignation

"come" is used to signify a speaker's annoyance or anger. While seemingly it might resemble the combination of "come" with a progressive verb, which is familiar in

Standard English, in AAVE, it has unique semantic-pragmatic role, meaning the speaker's indignation or disapproval about an action or event[4, 8, 11, 13].

"He come walkin' in here like he owned the damn place"[13]

"They come talkin' that trash"[13]

Use of "finna" to indicate immediate future actions

"finna" is a marker to indicate imminent actions or events. Derived potentially from the southern expression "fixin' to" and its variants like "fixta," "fitna," or "fidda," it denotes an immediate upcoming planned event[4, 8, 11, 13].

"I finna do it"[13]

"He finna go"[11]

3. Phonological Features

This research doesn't encompass the majority of phonological features due to LLM's inability to generate sound. However, we have included certain features that may also be reflected in writing.

Replacement of final "ing" with "in' "

The "-ing" ending is frequently pronounced and occasionally written as "-in'", omitting the final "g"[10, 12].

"He's runnin' fast"[4]

Omission of unstressed syllables at the beginning and middle

Unstressed initial and medial syllables tend to be deleted in speech, and sometimes this deletion is even reflected in their writing[11].

"'fraid"(afraid)

"Sec't'ry"(secretary)[11]

Though the lists provided here aren't comprehensive and might not cover every nuance of AAVE, we've endeavored to include its core characteristics and vital elements. As the cultural and societal landscape shifts, Urban AAVE is likely to adapt and evolve in response. Yet, considering its deep cultural significance, it's anticipated to continue standing out as a central and distinctive version of American English.

References

[1] Alim, H. S. (2004). *You know my steez : an ethnographic and sociolinguistic study of styleshifting in a Black American speech community* (Publication No. 3111683) [Doctoral dissertation, Stanford University]. ProQuest Dissertations & Theses.

[2] Baugh, J. (1986). A REEXAMINATION OF THE BLACK ENGLISH COPULA. In H. B. Allen & M. D. Linn (Eds.), *Dialect and Language Variation* (pp. 474–499). Academic Press. https://doi.org/10.1016/B978-0-12-051130-3.50040-9

[3] Baugh, J. (1983). *Black street speech : its history, structure, and survival* (1st ed.). University of Texas Press.

[4] Charity, A. H. (2008). African American English: An Overview. *Perspectives on Communication Disorders and Sciences in Culturally and Linguistically Diverse (CLD) Populations*, *15*(2), 33–42. https://doi.org/10.1044/cds15.2.33

[5] Cheshire, J. (1981). Variation in the Use of ain't in an Urban British English Dialect. *Language in Society*, *10*(3), 365–381. http://www.jstor.org/stable/4167261

[6] Dayton, E. (1996). *Grammatical categories of the verb in African-American Vernacular English*. University of Pennsylvania. https://search.proquest.com/openview/f9b1109e362eb7f7a70cf00c903b15aa/1?pq-origsite=gscholar&cbl=18750&diss=y

[7] Katz, S. R. (2020). *American English Grammar*. Routledge.

[8] Mufwene, I. S., Rickford, J., Baugh, J., & Bailey, G. (1998). COEXISTENT SYSTEMS IN AFRICAN-AMERICAN ENGLISH. *The Structure or African-Amreican English*, 110–153.

[9] Myhill, J. (1995). The Use of Features of Present-Day AAVE in the Ex-Slave Recordings. *American Speech*, *70*(2), 115–147. https://doi.org/10.2307/455812

[10] Pullum, G. K. (1999). African American Vernacular English is not standard English with mistakes. The Workings of Language: From Prescriptions to Perspectives, 59–66.

[11] Rickford, J. R. (1999). African American vernacular English : features, evolution, educational implications. Blackwell Publishers.

[12] Thomas, E. R. (2007). Phonological and phonetic characteristics of African American Vernacular English. *Language and Linguistics Compass, 1*, 450–475. https://doi.org/10.1111/j.1749-818X.2007.00029.x

[13] Wolfram, W. (2004). The grammar of urban African American vernacular English. *Handbook of varieties of English*, *2*, 111-32.

[14] Yule, G. (2014). *The study of language* (5th ed.). Cambridge university press. https://books.google.com/books?hl=en&lr=&id=mhOUEAAAQBAJ&oi=fnd&pg=PR11&dq=yule+2014+The+study+of+language+5th&ots=H0NKJqky4J&sig=ltufCX8dOLkyUmcZMnF4QMSO5iM
