# Supplementary material for: Enhancing Patient-Physician Communication: Simulating African American Vernacular English in Medical Diagnostics with Large Language Models
Source: J Healthc Inform Res. 2025 Mar 11;9(2):119–53. doi: 10.1007/s41666-025-00194-9 (PMC12037967; doi:10.1007/s41666-025-00194-9)
Supplement: Supplementary file 3 — Supplementary file3 (PDF 116 KB) [file 41666_2025_194_MOESM3_ESM.pdf]

Appendix 3

Medical Cases

1. Tension Pneumothorax
    a. Case Introduction
        i. A 65-year-old man is brought to the emergency department because of sharp chest pain and respiratory distress. He is in acute distress, moaning, and holding his hands over the right side of his chest.
    b. Initial vital signs
        i. Temperature: 37.0 degrees C (98.6 degrees F)
        ii. Pulse: 120 beats/min, Weak
        iii. Respiratory rate: 34 /minute
        iv. Blood pressure, systolic: 90 mm Hg
        v. Blood pressure, diastolic: 60 mm Hg
        vi. Height: 183 cm (72 in)
        vii. Weight: 97.5 kg (215 lb)
        viii. Body mass index: 29.1 kg/m2
    c. Initial history
        i. Reason(s) for visit: Chest pain; respiratory distress
        ii. History of Present illness:
            1. The patient, a 65-year-old man, is brought to the emergency department by ambulance from the trucking company where he works. About 10 minutes before the ambulance arrived, the patient developed excruciating, sharp pain in the right side of his chest and marked respiratory distress. He rates the pain as an 8 on a 10—point scale. The pain increases with respiration. He is unable to answer questions. A coworker who accompanied the patient to the hospital says that this never happened before, but the patient has had emphysema and asthma for years. Oxygen was administered during transport. All other history unobtainable.
2. Rheumatoid Arthritis
    a. Case Introduction
        i. A 32-year-old woman comes to the office because of increasing pain and swelling of her knees during the past week. She is well developed, well nourished, and in no apparent distress.
    b. Initial vital signs
        i. Temperature: 37.0 degrees C (98.6 degrees F)
        ii. Pulse: 65 beats/min (Regular rhythm)

iii. Respiratory rate: 16 /minute

iv. Blood pressure, systolic: 120 mm Hg

v. Blood pressure, diastolic: 75 mm Hg

vi. Height: 160 cm (63 in)

vii. Weight: 55.0 kg (121.3 lb)

viii. Body mass index: 21.5 kg/m2

c. Initial history

i. Reason(s) for visit: Knee pain; swelling

ii. History of present illness:

1. The patient, a 32-year-old woman, has experienced increasing fatigue and generalized weakness during the past 4 months. During the past 8 weeks, the patient has had generalized aches and joint stiffness most notably when she gets out of bed in the morning. The stiffness lasts 1 to 2 hours and makes it difficult to send the older children off to school. She also has had pain and intermittent swelling of the wrists and hands for approximately 4 weeks. She rates the pain as a 5 on a 10-point scale. Her knees have been swollen for the past 5 days. The pain and stiffness of her joints interfere with caring for her family. Acetaminophen provides minimal pain relief, to a 4 on a 10-point scale. There has been no fever or night sweats, and the patient has had no known infectious exposures. She has experienced decreased libido for 4 months.

iii. Past Medical History:

1. Hospitalization/Procedures: Childbirth at ages 31, 26, and 22
2. Other medical problems: None
3. Current medications: Oral contraceptive
4. Allergies: None
5. Vaccinations: Up to date

iv. Family History

1. Father, age 57, has hypertension
2. Mother, age 50, has type 2 diabetes mellitus
3. No siblings

v. Social History

1. Marital/Family: Married; three children
2. Sexual history: Active with spouse only; no history of sexually transmitted infections
3. Identifies as: African American
4. Personal habits: Does not smoke or drink alcoholic beverages; uses no

other medications or substances

5. Occupational/Educational: Small business owner; high school graduate
6. Recreational: Sings in church choir; baking

vi. Review of systems:

1. General: See HPI
2. Skin: Negative
3. HEENT: Negative
4. Musculoskeletal: See HPI
5. Cardiorespiratory: Negative
6. Gastrointestinal: Negative
7. Genitourinary: Gravida 3, para 3; menarche age 12; menses regular; last withdrawal bleeding 1 week ago; last cervical cytology 2 years ago; no history of abnormal cervical cytology
8. Neuropsychiatric: Negative

3. Ascending Aortic Dissection

a. Case Introduction

i. A 65-year-old woman comes to the emergency department because of severe left—sided chest pain. She appears anxious and diaphoretic.

b. Initial vital signs

i. Temperature: 37.0 degrees C (98.6 degrees F)

ii. Pulse: 110 beats/min (Bounding)

iii. Respiratory rate: 22 /minute

iv. Blood pressure, systolic: 190 mm Hg

v. Blood pressure, diastolic: 90 mm Hg

vi. Height: 168 cm (66.1 in)

vii. Weight: 65.0 kg (143.3 lb)

viii. Body mass index: 23.0 kg/m2

c. Initial history

i. Reason (s) for visit: Chest pain

ii. History of present illness:

1. The patient, a 65-year-old woman, comes to the emergency department because of the abrupt onset of sharp, left—sided chest pain 45 minutes ago. She rates the pain as an 8 on a 10—point scale. The pain radiates

to her left jaw and to her back. She is mildly short of breath, but the pain is not made worse by breathing or by a change in body position. She feels mildly nauseated but has no palpitations or light-headedness. She has had no fever, chills, cough, or hemoptysis. The patient was diagnosed with hypertension 5 years ago and has since been treated With lisinopril and hydrochlorothiazide combination. She has been adherent to her medication regimen and has not had any previous episodes of chest pain either at rest or on exertion.

iii. Past Medical History:

1. Hospitalizations/Procedures: Cesarean deliveries at ages 32 and 27
2. Other medical problems: None
3. Current medications: Lisinopril and hydrochlorothiazide
4. Allergies: None
5. Vaccinations: Up to date

d. Family History

i. Father died of a stroke at age 70

ii. Mother died of "natural causes" at age 92

iii. One brother, age 72, has hypertension

iv. Sisters, ages 69 and 55, are healthy

e. Social History:

i. Marital/Family: widowed 7 years ago; two adult children

ii. Sexual history: Not active since spouse's death; no history Of sexually transmitted infections

iii. Identifies as: White

iv. Personal habits: Does not smoke; drinks one glass of wine daily with dinner; uses no other medications or substances

v. Occupational/Educational: Retired insurance underwriter; high school graduate

vi. Recreational: Tournament bridge player; walks 2 to 3 miles daily

f. Review of systems:

i. General: See HPI

ii. Skin: Negative

iii. HEENT: Negative

iv. Musculoskeletal: Negative

v. Cardiorespiratory: See HPI

vi. Gastrointestinal: See HPI

vii. Genitourinary: Gravida 2, para 2; menopause age 50; no history of abnormal

cervical cytology

viii. Neuropsychiatric: Negative

4. Asthma

    a. Case Introduction

        i. A 4-year-old boy is brought to the office by his mother because of increasing shortness of breath and wheezing during the past 3 days. He appears well developed and in mild respiratory distress.

    b. Initial vital signs

        i. Temperature: 36.6 degrees C (97.9 degrees F)

        ii. Pulse: 116 beats/min (Regular rhythm)

        iii. Respiratory rate: 32 /minute

        iv. Blood pressure, systolic: 102 mm Hg

        v. Blood pressure, diastolic: 64 mm Hg

        vi. Height: 104 cm (40.9 in)

        vii. Weight: 18.0 kg (39.7 lb)

        viii. Body mass index: 16.6 kg/m2

    c. Initial history

        i. Reason (s) for visit: Shortness of breath; wheezing

        ii. History of present illness:

            1. The patient, a 4-year-old child, is brought to the office by his mother because of increasing shortness of breath during the past 3 days. The mother says her child has also been wheezing and has a cough that is worsening. He seems to cough more after playing outside. During the past year, the mother has noticed that he frequently wheezes after playing in the backyard. This usually resolves shortly after coming inside. As an infant, the patient had frequent episodes of "wheezy bronchitis' and numerous ear infections. He has had no recent fever, chills, earache, swollen glands, or sore throat. He is not coughing up mucus. The patient was hospitalized for 1 week at age 2 because of similar symptoms. During that hospitalization, he received intravenous (IV) antibiotics and oxygen by face mask.

        iii. Past Medical History:

            1. Hospitalization/Procedures: Shortness of breath and cough at age 2; pressure equalization tubes inserted at age 18 months

            2. Other medical problems: None

            3. Current medications: Steroid cream for atopic dermatitis

            4. Allergies: Pollen causes watery eyes and itching

            5. Vaccinations: Up to date

iv. Developmental History
    1. Age-appropriate milestones achieved
v. Family History:
    1. Father, age 36, and mother, age 30, healthy
    2. Sister, age 3, also healthy
vi. Social History
    1. Identifies as: Family identifies as white
vii. Review of systems:
    1. General: See HPI
    2. Skin: Atopic dermatitis
    3. HEENT: See HPI
    4. Musculoskeletal: Negative
    5. Cardiorespiratory: See HPI
    6. Gastrointestinal: Negative
    7. Genitourinary: Negative
    8. Neuropsychiatric: Negative

5. Diabetes with ketoacidosis; E. coli sepsis
    a. Case Introduction
        i. A 31-year-old woman is brought to the emergency department by her roommate because she has had nausea and vomiting and now is drowsy and lethargic. She appears acutely ill and is lying down in a fetal position.
    b. Initial vital signs
        i. Temperature: 38.1 degrees C (100.6 degrees F)
        ii. Pulse: 130 beats/min (Thready)
        iii. Respiratory rate: 26 /minute
        iv. Blood pressure, systolic: 90 mm Hg
        v. Blood pressure, diastolic: 70 mm Hg
        vi. Height: 173 cm (68.1 in)
        vii. Weight: 59.0 kg (130.1 lb)
        viii. Body mass index: 19.7 kg/m2
    c. Initial history
        i. Reason(s) for visit: Lethargy; nausea; vomiting
        ii. History of present illness:

1. The patient, a 31—year—old woman, is brought to the emergency department by her roommate because she has had nausea and vomiting for the past 24 hours. She became drowsy and lethargic during the past hour. She did not eat anything yesterday because of the nausea and vomiting. The patient has type 1 diabetes mellitus and usually takes insulin multiple times daily. She has not had any insulin during the past 24 hours. The roommate is unaware of the patient having any abdominal pain, diarrhea, or fever, although the patient said she had some chills yesterday. The patient rarely drinks alcoholic beverages and uses no other medications or substances.

iii. Past Medical History

1. Aside from diabetes, the roommate is not aware of other past illness; the patient usually takes insulin multiple times daily; all other history unobtainable.

iv. Family History: unobtainable

v. Social History

1. Marital/Family: Divorced; no children
2. Sexual history: Unobtainable
3. Identifies as: Mexican
4. Personal habits: Has smoked a half pack of cigarettes daily for 3 years; rarely drinks alcoholic beverages; uses no other medications or substances.
5. Occupational/Educational: Advertising sales representative; all other history unobtainable
6. Recreational: unobtainable

vi. Review of systems: unobtainable

6. Eclampsia

a. Case Introduction

i. A 25-year-old woman at 38 weeks' gestation is brought to the emergency department by her husband because of a seizure and a loss of consciousness. She is now conscious but appears confused.

b. Initial vital signs

i. Temperature: 37.2 degrees C (99 degrees F)

ii. Pulse: 112 beats/min (Regular rhythm; bounding)

iii. Respiratory rate: 16 /minute

iv. Blood pressure, systolic: 188 mm Hg

v. Blood pressure, diastolic: 114 mm Hg

vi. Height: 158 cm (62.2 in)

vii. Weight: 65.5 kg (144.4 lb)

viii. Body mass index: 26.2 kg/m2

c. Initial history

i. Reason(s) for visit: seizure; loss of consciousness

ii. History of present illness:

1. The patient, a 25-year-old woman at 38 weeks' gestation, is brought to the emergency department by her husband after he found her having a seizure 10 minutes ago. The seizure lasted only a few minutes. The patient is gravida 1, para 0. She has been receiving routine prenatal care and thus far has had an uncomplicated pregnancy. The husband says she had a severe headache for the past 3 days, and her feet appeared swollen for the past 2 to 3 weeks. The patient has never had a seizure before and has never been treated for any renal or neurologic disease. She has no history of hypertension. The patient's last prenatal visit 2 weeks ago was uneventful. The patient is conscious but appears confused.

iii. Past Medical History:

1. Hospitalization/Procedures: None
2. Other medical problems: None
3. Current medications: Prenatal vitamins
4. Allergies: None
5. Vaccinations: Up to date

iv. Family History

1. Father, age 55, healthy
2. Mother, age 52, had endometrial cancer 2 years ago
3. No siblings

v. Social History

1. Marital/Family: Married; no children
2. Sexual history: Active with spouse only; no history of sexually transmitted infections
3. Identifies as: White
4. Personal habits: Does not smoke or drink alcoholic beverages; uses no other medications or substances
5. Occupational/Educational: Nursing assistant; high school graduate
6. Recreational: Horseback riding; reading; movies

vi. Review of systems:

1. General: See HPI

2. Skin: Negative
3. HEENT: Negative
4. Musculoskeletal: See HPI
5. Cardiorespiratory: Negative
6. Gastrointestinal: Negative
7. Genitourinary: See HPI; Gravida 1, para 0; menarche age 13; last menstrual period 38 weeks ago was normal; last cervical cytology 16 months ago
8. Neuropsychiatric: See HPI
