# Supplementary material for: Enhancing Patient-Physician Communication: Simulating African American Vernacular English in Medical Diagnostics with Large Language Models
Source: J Healthc Inform Res. 2025 Mar 11;9(2):119–53. doi: 10.1007/s41666-025-00194-9 (PMC12037967; doi:10.1007/s41666-025-00194-9)
Supplement: Supplementary file 4 — Supplementary file4 (PDF 162 KB) [file 41666_2025_194_MOESM4_ESM.pdf]

Appendix 4

Comprehensive Statistical Results

⁎ For *P* value for one-tailed test, the hypothesis is that Group 2 > Group 1

Table 1. All Types

	GPT-4			Llama 3		
	**Count**	**Sum**	**Mean (SD)**	**Count**	**Sum**	**Mean (SD)**
**DemoP**	36	300	8.333 (3.964)	36	414	11.500 (3.418)
**LingP**	36	249	6.917 (4.211)	36	495	13.750 (4.753)
**CompP**	36	354	9.833 (4.411)	36	578	16.056 (5.909)
	**Sum of Squares**	***F (2, 70)***	***P*-value**	**Sum of Squares**	***F (2, 70)***	***P*-value**
**Condition**	153.167	6.218	0.003	373.574	12.124	<0.001
**Error**	862.167			1078.426		

1-1 One-Way Repeated Measures ANOVA

	GPT-4			Llama 3		
**Group1**	**DemoP**	**DemoP**	**LingP**	**DemoP**	**DemoP**	**LingP**
**Group2**	**LingP**	**CompP**	**CompP**	**LingP**	**CompP**	**CompP**
**Mean**	-1.417	1.500	2.917	2.250	4.556	2.306
**SD**	4.965	4.681	5.228	5.432	5.739	5.476
**SE**	0.827	0.780	0.871	0.905	0.957	0.913
***t* test (*df=35*)**	-1.712	1.923	3.347	2.485	4.762	2.526
***P* (1-tail)**	0.952	0.031	<0.001	0.009	<0.001	0.008
***P* (2-tail)**	0.096	0.063	0.002	0.018	<0.001	0.016

1-2 Paired *t*-Test

Table 2. Pre-verbal markers

	GPT-4			Llama 3		
	**Count**	**Sum**	**Mean (SD)**	**Count**	**Sum**	**Mean (SD)**
**DemoP**	36	35	0.972 (1.028)	36	19	0.528 (0.941)
**LingP**	36	52	1.444 (1.229)	36	119	3.306 (2.638)
**CompP**	36	68	1.889 (1.753)	36	100	2.778 (2.231)
	**Sum of Squares**	***F (2, 70)***	***P*-value**	**Sum of Squares**	***F (2, 70)***	***P*-value**
**Condition**	15.130	7.334	0.001	156.685	22.477	<0.001
**Error**	72.204			243.981		

2-1 One-Way Repeated Measures ANOVA

	GPT-4			Llama 3		
**Group1**	**DemoP**	**DemoP**	**LingP**	**DemoP**	**DemoP**	**LingP**
**Group2**	**LingP**	**CompP**	**CompP**	**LingP**	**CompP**	**CompP**
**Mean**	0.472	0.917	0.444	2.778	2.250	-0.528
**SD**	1.230	1.461	1.594	2.819	2.465	2.624
**SE**	0.205	0.244	0.266	0.470	0.411	0.437
***t* test (*df=35*)**	2.303	3.763	1.673	5.911	5.476	-1.207
***P* (1-tail)**	0.014	<0.001	0.052	<0.001	<0.001	0.882
***P* (2-tail)**	0.027	<0.001	0.103	<0.001	<0.001	0.236

2-2 Paired *t*-Test

Table 3. Verbal tense-number marking

	GPT-4			Llama 3		
	Count	Sum	Mean (SD)	Count	Sum	Mean (SD)
**DemoP**	36	7	0.194 (0.401)	36	5	0.139 (0.424)
**LingP**	36	3	0.083 (0.280)	36	28	0.778 (0.898)
**CompP**	36	5	0.139 (0.424)	36	23	0.639 (0.931)
	**Sum of Squares**	***F (2, 70)***	***P*-value**	**Sum of Squares**	***F (2, 70)***	***P*-value**
**Condition**	0.222	1.094	0.341	8.130	10.209	<0.001
**Error**	7.111			27.870		

3-1 One-Way Repeated Measures ANOVA

	GPT-4			Llama 3		
**Group1**	**DemoP**	**DemoP**	**LingP**	**DemoP**	**DemoP**	**LingP**
**Group2**	**LingP**	**CompP**	**CompP**	**LingP**	**CompP**	**CompP**
**Mean**	-0.111	-0.056	0.056	0.639	0.500	-0.139
**SD**	0.465	0.475	0.410	0.723	0.971	0.961
**SE**	0.077	0.079	0.068	0.121	0.162	0.160
***t* test (*df=35*)**	-1.435	-0.702	0.813	5.301	3.090	-0.867
***P* (1-tail)**	0.920	0.756	0.211	<0.001	0.002	0.804
***P* (2-tail)**	0.160	0.487	0.422	<0.001	0.004	0.392

3-2 Paired *t*-Test

Table 4. Nouns and pronouns

	GPT-4			Llama 3		
	**Count**	**Sum**	**Mean (SD)**	**Count**	**Sum**	**Mean (SD)**
**DemoP**	36	1	0.028 (0.167)	36	17	0.472 (0.736)
**LingP**	36	3	0.083 (0.368)	36	25	0.694 (0.920)
**CompP**	36	10	0.278 (0.513)	36	31	0.861 (0.990)
	**Sum of Squares**	***F (2, 70)***	***P*-value**	**Sum of Squares**	***F (2, 70)***	***P*-value**
**Condition**	1.241	4.958	0.010	2.741	2.575	0.083
**Error**	8.759			37.259		

4-1 One-Way Repeated Measures ANOVA

	GPT-4			Llama 3		
**Group1**	**DemoP**	**DemoP**	**LingP**	**DemoP**	**DemoP**	**LingP**
**Group2**	**LingP**	**CompP**	**CompP**	**LingP**	**CompP**	**CompP**
**Mean**	0.056	0.250	0.194	0.222	0.389	0.167
**SD**	0.410	0.500	0.577	1.017	0.934	1.134
**SE**	0.068	0.083	0.096	0.170	0.156	0.189
***t* test (*df=35*)**	0.813	3.000	2.023	1.311	2.497	0.882
***P* (1-tail)**	0.211	0.002	0.025	0.099	0.009	0.192
***P* (2-tail)**	0.422	0.005	0.051	0.199	0.017	0.384

4-2 Paired *t*-Test

Table 5. Negation

	GPT-4			Llama 3		
	**Count**	**Sum**	**Mean (SD)**	**Count**	**Sum**	**Mean (SD)**
**DemoP**	36	101	2.806 (1.546)	36	64	1.778 (1.333)
**LingP**	36	84	2.333 (1.568)	36	78	2.167 (1.320)
**CompP**	36	113	3.139 (1.376)	36	90	2.500 (1.444)
	**Sum of Squares**	***F (2, 70)***	***P*-value**	**Sum of Squares**	***F (2, 70)***	***P*-value**
**Condition**	11.796	3.553	0.034	9.407	4.494	0.015
**Error**	116.204			73.259		

5-1 One-Way Repeated Measures ANOVA

	GPT-4			Llama 3		
**Group1**	**DemoP**	**DemoP**	**LingP**	**DemoP**	**DemoP**	**LingP**
**Group2**	**LingP**	**CompP**	**CompP**	**LingP**	**CompP**	**CompP**
**Mean**	-0.472	0.333	0.806	0.389	0.722	0.333
**SD**	1.748	1.927	1.786	1.536	1.344	1.454
**SE**	0.291	0.321	0.298	0.256	0.224	0.242
***t* test (*df=35*)**	-1.621	1.038	2.706	1.519	3.224	1.375
***P* (1-tail)**	0.943	0.153	0.005	0.069	0.001	0.089
***P* (2-tail)**	0.114	0.307	0.010	0.138	0.003	0.178

5-2 Paired *t*-Test

Table 6. Lexical features

	GPT-4			Llama 3		
	**Count**	**Sum**	**Mean (SD)**	**Count**	**Sum**	**Mean (SD)**
**DemoP**	36	1	0.028 (0.167)	36	0	0.000 (0.000)
**LingP**	36	0	0.000 (0.000)	36	4	0.111 (0.319)
**CompP**	36	0	0.000 (0.000)	36	7	0.194 (0.401)
	**Sum of Squares**	***F (2, 70)***	***P*-value**	**Sum of Squares**	***F (2, 70)***	***P*-value**
**Condition**	0.019	1.000	0.373	0.685	4.512	0.014
**Error**	0.648			5.315		

6-1 One-Way Repeated Measures ANOVA

	GPT-4			Llama 3		
**Group1**	**DemoP**	**DemoP**	**LingP**	**DemoP**	**DemoP**	**LingP**
**Group2**	**LingP**	**CompP**	**CompP**	**LingP**	**CompP**	**CompP**
**Mean**	-0.028	-0.028	0.000	0.111	0.194	0.083
**SD**	0.167	0.167	0.000	0.319	0.401	0.439
**SE**	0.028	0.028	0.000	0.053	0.067	0.073
***t* test (*df=35*)**	-1.000	-1.000	N/A	2.092	2.907	1.139
***P* (1-tail)**	0.838	0.838	N/A	0.022	0.003	0.131
***P* (2-tail)**	0.324	0.324	N/A	0.044	0.006	0.263

6-2 Paired *t*-Test

Table 7. Phonological features

	GPT-4			Llama 3		
	**Count**	**Sum**	**Mean (SD)**	**Count**	**Sum**	**Mean (SD)**
**DemoP**	36	68	1.889 (1.833)	36	245	6.806 (3.520)
**LingP**	36	32	0.889 (1.214)	36	200	5.556 (2.273)
**CompP**	36	86	2.389 (1.946)	36	249	6.917 (3.557)
	**Sum of Squares**	***F (2, 70)***	***P*-value**	**Sum of Squares**	***F (2, 70)***	***P*-value**
**Condition**	42.000	8.681	<0.001	41.130	3.097	0.051
**Error**	169.333			464.870		

7-1 One-Way Repeated Measures ANOVA

	GPT-4			Llama 3		
**Group1**	**DemoP**	**DemoP**	**LingP**	**DemoP**	**DemoP**	**LingP**
**Group2**	**LingP**	**CompP**	**CompP**	**LingP**	**CompP**	**CompP**
**Mean**	-1.000	0.500	1.500	-1.250	0.111	1.361
**SD**	2.330	2.063	2.197	3.850	3.831	3.217
**SE**	0.388	0.344	0.366	0.642	0.638	0.536
***t* test (*df=35*)**	-2.575	1.454	4.096	-1.948	0.174	2.538
***P* (1-tail)**	0.993	0.077	<0.001	0.970	0.431	0.008
***P* (2-tail)**	0.014	0.155	<0.001	0.059	0.863	0.016

7-2 Paired *t*-Test

Table 8. Out of list

	GPT-4			Llama 3		
	**Count**	**Sum**	**Mean (SD)**	**Count**	**Sum**	**Mean (SD)**
**DemoP**	36	87	2.417 (1.251)	36	64	1.778 (0.866)
**LingP**	36	75	2.083 (1.795)	36	41	1.139 (0.867)
**CompP**	36	72	2.000 (1.586)	36	78	2.167 (1.384)
	**Sum of Squares**	***F (2, 70)***	***P*-value**	**Sum of Squares**	***F (2, 70)***	***P*-value**
**Condition**	3.500	0.814	0.447	19.389	8.418	<0.001
**Error**	150.500			80.611		

8-1 One-Way Repeated Measures ANOVA

	GPT-4			Llama 3		
**Group1**	**DemoP**	**DemoP**	**LingP**	**DemoP**	**DemoP**	**LingP**
**Group2**	**LingP**	**CompP**	**CompP**	**LingP**	**CompP**	**CompP**
**Mean**	-0.333	-0.417	-0.083	-0.639	0.389	1.028
**SD**	2.111	2.005	2.103	1.150	1.661	1.682
**SE**	0.352	0.334	0.350	0.192	0.277	0.280
***t* test (*df=35*)**	-0.947	-1.247	-0.238	-3.333	1.405	3.667
***P* (1-tail)**	0.825	0.890	0.593	0.999	0.084	<0.001
***P* (2-tail)**	0.350	0.221	0.813	0.002	0.169	<0.001

8-2 Paired *t*-Test
